# Environmental DNA metabarcoding to detect pathogenic *Leptospira* and associated organisms in leptospirosis-endemic areas of Japan

**DOI:** 10.1038/s41598-019-42978-1

**Published:** 2019-04-25

**Authors:** Yukuto Sato, Masaru Mizuyama, Megumi Sato, Toshifumi Minamoto, Ryosuke Kimura, Claudia Toma

**Affiliations:** 1 Center for Strategic Research Project, Organization for Research Promotion, University of the Ryukyus, 1 Senbaru, Nishihara, Okinawa 903-0213 Japan; 2 Department of Bacteriology, Graduate School of Medicine, University of the Ryukyus, 207 Uehara, Nishihara, Okinawa 903-0215 Japan; 3 Graduate School of Health Sciences, Niigata University, 2-746 Asahimachi-dori, Chuo-ku, Niigata 951-8122 Japan; 4 Graduate School of Human Development and Environment, Kobe University, 3-11 Tsurukabuto, Nada-ku, Kobe 657-8501 Japan; 5 Department of Human Biology and Anatomy, Graduate School of Medicine, University of the Ryukyus, 207 Uehara, Nishihara, Okinawa 903-0215 Japan; 6 Present Address: Graduate School of Engineering and Science, University of the Ryukyus, 1 Senbaru, Nishihara, Okinawa 903-0213 Japan

**Keywords:** Microbial ecology, Microbiology, Microbial ecology, Microbiology, Microbial ecology

## Abstract

Leptospires, which cause the zoonotic disease leptospirosis, persist in soil and aqueous environments. Several factors, including rainfall, the presence of reservoir animals, and various abiotic and biotic components interact to influence leptospiral survival, persistence, and pathogenicity in the environment. However, how these factors modulate the risk of infection is poorly understood. Here we developed an approach using environmental DNA (eDNA) metabarcoding for detecting the microbiome, vertebrates, and pathogenic *Leptospira* in aquatic samples. Specifically, we combined 4 sets of primers to generate PCR products for high-throughput sequencing of multiple amplicons through next-generation sequencing. Using our method to analyze the eDNA of leptospirosis-endemic areas in northern Okinawa, Japan, we found that the microbiota in each river shifted over time. Operating taxonomic units corresponding to pathogenic *L*. *alstonii*, *L*. *kmetyi*, and *L*. *interrogans* were detected in association with 12 nonpathogenic bacterial species. In addition, the frequencies of 11 of these species correlated with the amount of rainfall. Furthermore, 10 vertebrate species, including *Sus scrofa*, *Pteropus dasymallus*, and *Cynops ensicauda*, showed high correlation with leptospiral eDNA detection. Our eDNA metabarcoding method is a powerful tool for understanding the environmental phase of *Leptospira* and predicting human infection risk.

## Introduction

Leptospirosis is a zoonotic disease that is caused by species of the spirochete genus *Leptospira*. Humans become infected through contact with the urine of infected reservoir animals or contaminated water or soil^1^. Pathogenic *Leptospira* can colonize the kidneys of diverse vertebrates, including rodents, cattle, wild boars, and dogs^1^. In addition, flooding, occupational activities, and recreational pursuits are risk factors associated with *Leptospira* infection in humans^2^. Leptospirosis is endemic in Okinawa, the southernmost prefecture of Japan, where infection occurs predominantly after freshwater exposure^3,4^. Sporadic cases and outbreaks of leptospirosis after recreational activities in rivers have been reported mainly in the northern part of Okinawa Island and the Yaeyama region^5–7^. Outbreaks among US military personnel have also been reported several times in Northern Okinawa, where the Jungle Warfare Training Center operates^8^.

A pathogenic *Leptospira* species (*L*. *kmetyi*) has recently been isolated from the Okinawan environment^9^. In contrast, *L*. *interrogans*, the main pathogenic species recovered from clinical patients, has not yet been isolated from the environment. Environmental DNA (eDNA) in aquatic environments originates from various sources, including feces, urine, damaged tissue, and microorganisms, and has successfully been used to identify fish, terrestrial animals, and eukaryotic pathogens in ponds, rivers, and seawater^10–13^. eDNA becomes an efficient tool for monitoring eukaryotes when mitochondrial genes such as 12S rRNA are targeted^10,13^. For bacterial detection, 16S rRNA gene partial fragments have been targeted for the analysis of environmental and human-associated microbiomes^14–17^. Environmental *Leptospira* DNA has been successfully barcoded by using 16S rRNA, *flaB*, *lipL32* or *secY* genes^18–22^. There is no report that attempt metabarcoding *Leptospira* and its surrounding macro/microbiome in the environment. However, there is one 16S metagenomic approach to detect *Leptospira* and other bacterial pathogens in rodents^23^.

The risk of infection after contact with *Leptospira*-contaminated environmental water depends on the ability of bacteria to survive, persist and infect new hosts^2^. Rain and various extreme weather events are considered primary risk factors for leptospirosis, and outbreaks after floods have been reported worldwide^24,25^. However, many other factors that favor the environmental cycling and transmission of *Leptospira* species are not well understood^2,26,27^. Pathogenic *Leptospira* can form biofilms and cell aggregation *in vitro* as well as *in vivo*, thus, these bacteria might use these strategies to circumvent the dilution of urine in large bodies of water and to maintain bacteria sufficiently concentrated to achieve infection^28–31^. The composition of microbial communities also influences the formation of biofilms, thus, the survival and persistence of infectious *Leptospira*^2^. Little to no information is available regarding the micro- and macroorganisms associated or co-existing with *Leptospira* that might support its survival outside the host.

In the current study, we hypothesized that eDNA from leptospirosis-endemic areas can be used to detect the presence of pathogenic *Leptospira* species and potential host organisms, as well as microbiomes associated with leptospiral persistence in the environment. To these ends, we developed a method for multiplex parallel DNA sequencing of metabarcoded PCR products as a tool for studying the environmental phase of *Leptospira* species.

## Results

### Multiplex environmental DNA metabarcoding for detecting *Leptospira* species

**Figure 1:**
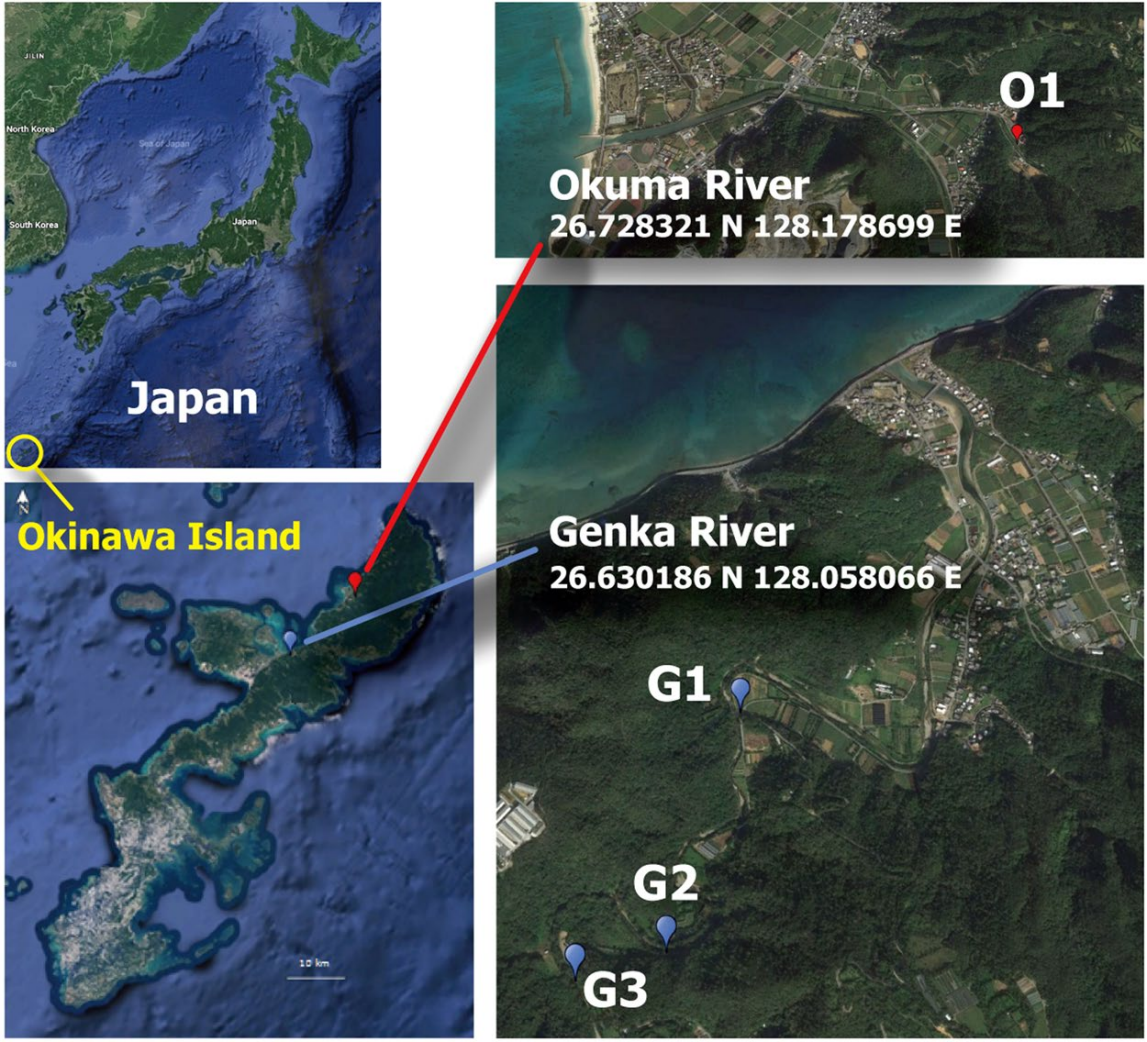
Sampling locations of river water. O1, Okuma-1 sampling site; and G1, G2, and G3, Genka-1, -2, and -3 sampling sites, respectively. Satellite imagery were obtained from Google Maps (https://www.google.com/maps/); data providers of the satellite imagery are Google, Data SIO, NOAA, U.S. Navy, NGA, GEBCO, Landsat/Copernicus, Data LDEO-Columbia, and NSF. Adobe Illustrator CS6 was used to create the map with satellite imagery.

To detect *Leptospira* species, related bacteria, and potential reservoir vertebrates through eDNA analysis, we collected 80 samples of river water from leptospirosis-endemic areas in Okinawa Island, Japan, and successfully extracted highly pure eDNA from 69 samples (Fig. 1 and Supplementary Fig. S1). From each of the Genka and Okuma Rivers, we collected ten samples (500 mL each) at each of four time points (July 10, August 7, September 11, and October 16, 2017). From each sample, we extracted 3.62 ± 0.45 ng/μL (mean ± S.E.) of eDNA with OD_260/280_ values of 2.22 ± 0.62 (Supplementary Table S1).

**Figure 2:**
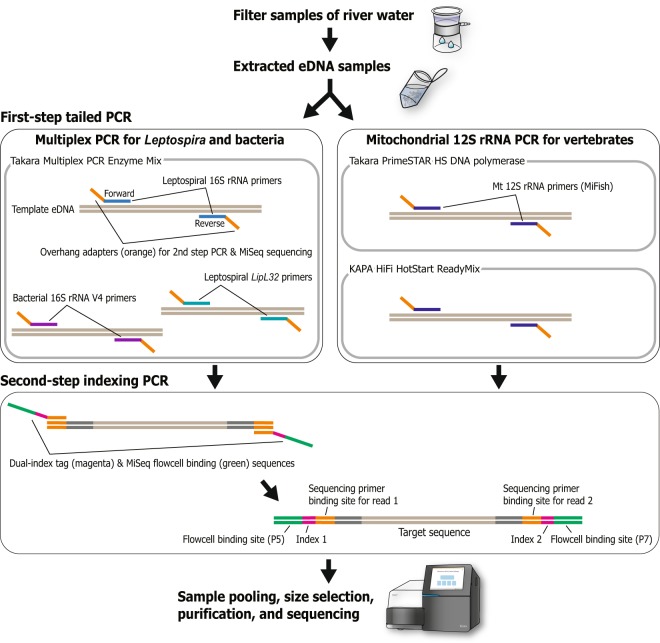
Schematic view of the procedure of library preparation for metabarcoding sequencing based on a two-step tailed PCR. Multiplex PCR was applied in the first step reaction for *Leptospira* and bacterial detection. The procedure for vertebrate mitochondrial 12S rRNA sequencing was basically the same but slightly modified from that of Miya *et al*.^10^.

From each eDNA sample, partial fragments of leptospiral 16S rRNA^21^ and *lipL32*^32^ genes and a broadly conserved bacterial 16S rRNA gene V4 region^15^ were amplified in a multiplex manner and sequenced by using the Illumina MiSeq platform (for details, see Materials and Methods). As an efficient, cost-effective method for *Leptospira* detection, we performed a single sequencing run on a pooled library of the described leptospiral and bacterial PCR products by using the MiSeq Micro flowcell (maximum, 5 million reads) (Fig. 2). This process produced 3,582,426 pairs of raw sequences with 44,780 ± 1,252 reads per sample. After primary data processing (see Materials and Methods), a total of 3,408,828 reads remained as quality-filtered sequences, with 42,610 ± 1,247 reads per sample. Among them, both primer ends of the amplified bacterial 16S rRNA V4 region were found in 3,400,844 reads (42,510 ± 1,244 reads per sample), those of leptospiral 16S rRNA were found in 381 reads (4.76 ± 0.91 reads per sample), and those of *lipL32* were found in 7,603 reads (95.04 ± 16.76 reads per sample).

**Figure 3:**
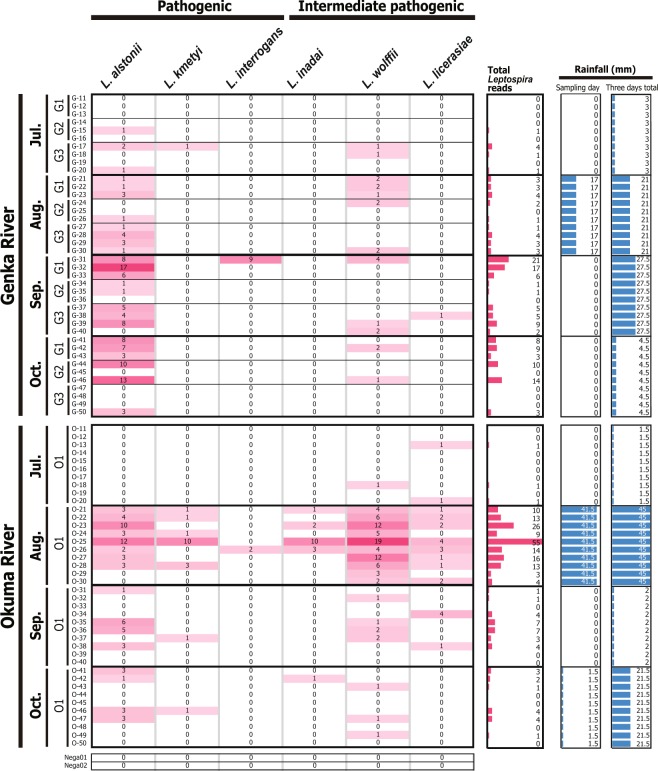
Environmental detection of pathogenic leptospiral 16S rRNA gene. Number of sequence reads detected in each sample are shown with colored matrices in pink shading. Pink bars indicate the total number of *Leptospira* reads per sample summed across species. Blue bars show the amount of rainfall (mm) on the sampling day (left column) and that comprising two days before sampling, the day before the sampling, and the day of sampling (right column). O1, G1, G2, and G3 indicates sampling locations Okuma-1, Genka-1, -2, and -3, respectively. G-11–G-50 and O-11–O-50 denote sample names.

From the 381 putative leptospiral 16S rRNA gene sequences, we found a total of 353 non-singleton (≥2 counts), true-positive sequences (pink shading in Fig. 3). The remaining 28 sequences were singletons in a sample (total, 10 reads) or reads with no hits in the Blast database (total, 18 reads). The species annotation of each sequence was achieved primarily through Blast-based analysis against the National Center for Biotechnology Information (NCBI) nucleotide collection database^33^ and carefully corrected on the basis of their molecular phylogeny as analyzed by using type sequences of the *Leptospira* 16S rRNA gene (Supplementary Fig. S2). Leptospiral 16S rRNA gene sequences were detected strongly in the samples from Okuma River in August (pink bars in Fig. 3; 163 reads in total), and similarly relatively strong signals were detected in the samples from Genka River during September and October (67 and 47 reads in total, respectively). In addition, at least 10 reads of pathogenic *Leptospira* species (*L*. *alstonii*, *L*. *kmetyi*, and *L*. *interrogans*) were detected in the September and October samples from Genka River and the August sample from Okuma River (Fig. 3, dark-pink shading).

From the 7,603 of putative leptospiral *lipL32* sequences, we obtained a total of 3,417 non-singleton, true-positive sequences (Supplementary Fig. S3). Their species annotation was achieved through Blast-based analysis against the NCBI nucleotide database^33^ and molecular phylogeny with known leptospiral *lipL32* sequences (Supplementary Fig. S4). Relatively strong signals again were detected for the August sample from the Okuma River (total, 1,815 reads), whereas sporadic signals were observed for all other months and locations (Supplementary Fig. S3, orange shading) except the July samples of Okuma River. The remaining 4,186 sequences (7,603–3,417) included reads with no Blast hits (total, 4,132 reads), those with a Blast hit to other bacteria (total, 40 reads; gray shading in Supplementary Fig. S3), and singletons in a sample (total, 14 reads).

### Correlation of *Leptospira* eDNA with environmental factors and other bacteria

The eDNA detection of *Leptospira* was positively correlated with the amount of rainfall (shown as blue bars in Fig. 3 and Supplementary Fig. S3). We considered each water sample from each location (river) and time point (month) to be statistically independent. Thus, we merged the number of detected leptospiral sequences (shown as pink bars in Fig. 3 and orange bars in Supplementary Fig. S3) according to the respective locations and months (2 rivers with 4 time points). The merged number of leptospiral sequences was significantly correlated with the amount of rainfall (mm) of the sampling day for both *Leptospira* 16S rRNA and *lipL32* genes (16S rRNA: *r* = 0.8283, *d*.*f*. = 6, *P* = 0.0111; *lipL32*: *r* = 0.8692, *d*.*f*. = 6, *P* = 0.0051). Even when the rainfall amounts for the 2 days before and the day before sampling were included, both types of reads were significantly correlated with rainfall (16S rRNA: *r* = 0.8186, *d*.*f*. = 6, *P* = 0.0130; *lipL32*: *r* = 0.7488, *d*.*f*. = 6, *P* = 0.0325). In addition, when the 80 samples were treated as independent data points, the correlations remained significant (16S rRNA: *r* = 0.5257, *d*.*f*. = 78, *P* < 0.0001; *lipL32*: *r* = 0.4953, *d*.*f*. = 78, *P* < 0.0001). In contrast, no other environmental parameter, including temperature (°C), humidity (%), water temperature (°C), and weather at the time of sampling (sunny, cloudy, or rainy), was correlated with *Leptospira* eDNA detection (Supplementary Table S2).

**Figure 4:**
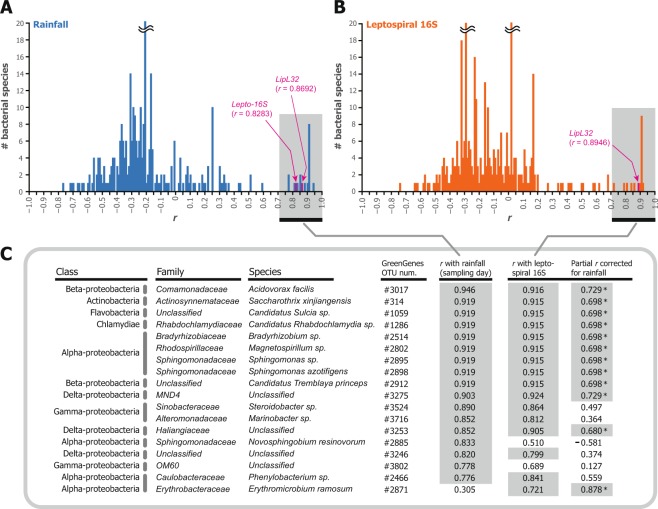
Correlation of bacterial 16S rRNA gene with rainfall and *Leptospira*. Histograms show distributions of Pearson’s correlation coefficient scores between the detected read numbers of 355 bacterial species and (**A**) rainfall amount on sampling day (mm) and (**B**) detected read number of leptospiral 16S rRNA gene. (**C**) Class, family, species, and operational taxonomic unit (OTU) numbers from GreenGenes database of 18 bacterial species that show significant correlation *r* (indicated by gray shading) with both or either rainfall amount and *Leptospira* detection (*P* < 0.05 and Benjamini–Hochberg-corrected false discovery rate < 0.05). The rightmost column indicates the partial correlations between each bacterium and *Leptospira* detection when controlled for rainfall; asterisks and gray shading denote correlations that remain significant at a level of *P* < 0.05.

In addition, 15 species of riverine bacteria were significantly correlated with *Leptospira* detection (Fig. 4). From the 3,400,844 putative bacterial 16S rRNA gene V4 sequences described earlier, we identified 1,018,384 reliable reads that were repeatedly sequenced 10 times or more (approximately 250 parts per million [ppm] on average) in at least one sample. Species annotation of these universal bacterial 16S rRNA sequences was achieved through Blast-based analysis against the GreenGenes database^34^, and a total of 355 bacterial species were obtained from 911,392 sequences (11,392 ± 659 sequences per sample; Supplementary Fig. S5). The remaining 106,992 (1,337 ± 152 sequences per sample) yielded no Blast hits. A non-metric multidimensional scaling plot of the standardized bacteriome of each sample (Supplementary Fig. S6) indicated that the two rivers (Genka and Okuma Rivers) had distinct bacterial microbiota, which roughly clustered according to time point (July through October). In contrast, among the 18 bacteria listed in Fig. 4C, 15 showed significant correlation with both rainfall amount and leptospiral 16S rRNA (*r* > 0.71, *d*.*f*. = 6, *P* < 0.05, Benjamini–Hochberg [BH]-corrected false discovery rate [FDR] <0.05; Fig. 4). Among these 15 bacterial species, 12 retained significant partial correlation with *Leptospira* detection when corrected for rainfall (indicated by asterisks on the right side of Fig. 4C). The majority of these potentially *Leptospira*-related bacteria were proteobacteria (α-, β-, γ-, and δ-proteobacteria); one species each of Actinobacteria, Flavobacteria, and intracellular bacteria Chlamydiae was included also.

### Environmental DNA detection of vertebrates correlated with *Leptospira*

**Figure 5:**
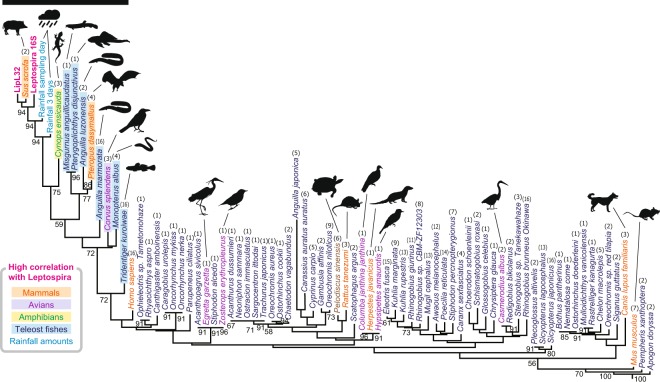
Neighbor-joining clustering according to a series of correlation coefficients between *Leptospira* and vertebrate eDNA detection. The clustering tree was generated based on Euclidean distances among a series of Pearson’s correlation coefficients between detected read numbers of *Leptospira* (results from 16S rRNA and *lipL32* genes) and those of the vertebrate mitochondrial 12S rRNA gene (results from KAPA-HiFi and PrimeStar-HS polymerases) or rainfall amount (mm; Supplementary Table S3). The black bar indicates 10 vertebrate species that showed significant correlation with leptospiral 16S rRNA gene detection (*P* < 0.05 and Benjamini–Hochberg-corrected false discovery rate < 0.05). Numbers in parentheses above scientific names denote the number of appearances of the species (maximum, 16 [i.e., 2 rivers multiplied by 4 months multiplied by 2 PCR-enzyme results]). Numbers on the tree indicate support values for the nodes estimated from 100 bootstrap replications.

To address the potential reservoir animals of *Leptospira* by using eDNA analysis, we amplified fragments of the mitochondrial 12 S rRNA genes of vertebrates^10^ and sequenced these products in another MiSeq run using a Nano flowcell (maximum, 1 million reads), thus generating 506,622 sequence reads) (Fig. 2). These were derived from PCR amplification of the 80 water samples by using the DNA polymerases KAPA-HiFi (175,872 total reads; 2,198 ± 361 reads per sample) and PrimeStar-HS (330,750 total reads; 4,134 ± 596 reads per sample) DNA (for details, see Materials and Methods). In total, 323,732 reads remained after primary data processing, and we identified 311,700 non-singleton, true-positive sequences (143,044 reads at 1,788 ± 345 reads per sample from KAPA-HiFi and 168,656 reads at 2,108 ± 512 reads per sample from PrimeStar amplicons). Species annotation by using the extended version of MiFish pipeline^12^ with tetrapod data from the NCBI nucleotide database^33^ yielded a total of 74 vertebrate species detected comprising 7 mammals, 6 avians, 1 reptile, 1 amphibian, and 59 teleost fish species (Fig. 5 and Supplementary Fig. S7).

We found that 10 vertebrate species showed high correlation with the eDNA detection of *Leptospira* (Fig. 5). These species included boar (*Sus scrofa*), fruit bat (*Pteropus dasymallus*), house crow (*Corvus splendens*), sword-tail newt (*Cynops ensicauda*), and riverine benthic fishes such as eels (*Anguilla marmorata* and *Anguilla luzonensis*) and oriental weather loach (*Misgurnus anguillicaudatus*). Among these vertebrates, boar, sword-tail newt, loach, invasive janitor fish (*Pterygoplichthys disjunctivus*), and eel (*A*. *luzonensis*) yielded particularly high correlation scores (*r* = 0.9153–0.9444, *d*.*f*. = 6, *P* = 0.0014–0.0004, BH-corrected FDR < 0.01). In addition, fruit bat and Asian swamp eel (*Monopterus albus*) demonstrated relatively high and significant correlations (*r* = 0.7311–0.8937, *d*.*f*. = 6, *P* = 0.0393–0.0028, BH-corrected FDR < 0.05; Supplementary Table S3). Correlation analysis of these vertebrates with the *Leptospira*-correlated bacteria presented in Fig. 4C highlighted the same 10 animal and fish species (Supplementary Fig. S8). When corrected for *Leptospira* detection, significant partial correlation with boar (*S*. *scrofa*) and eel (*A*. *marmorata*) remained for most of these bacteria (Supplementary Figs S9 and S10).

## Discussion

Because leptospiral survival and persistence in the environment rely on interactions with other microorganisms and reservoir hosts, we developed an eDNA-based approach to identify these factors and then used our method to analyze the eDNA from 2 leptospirosis-endemic rivers in Japan (Fig. 1). Using metabarcoding of the universal 16S rRNA gene V4 region enabled us to characterize the environmental microbiome, whereas primer sets specific for *Leptospira* detected the presence of pathogenic *(L*. *alstonii*, *L*. *kmetyi*, *L*. *interrogans*) and intermediate pathogenic (*L*. *inadai*, *L*. *wolfii*, *L*. *licerasiae*; Fig. 3 and Supplementary Fig. S3) *Leptospira* species. Universal PCR primers targeting the hypervariable V4 region of the 16S rRNA gene previously detected *Leptospira* in kidney samples of reservoir animals^22,35^. However, our results showed that when used on a complex environmental sample such as river water, these universal PCR primers detected the predominating bacterial species but not *Leptospira* species (Supplementary Fig. S5). In addition, *L*. *interrogans* was recently found to persist in soil and spring water microcosmos at concentrations that are below the limit of detection by qPCR analysis^36^, suggesting that *Leptospira* species in general survive in the environment at very low concentrations. Although *L*. *kmetyi* has been detected previously in the Okinawan environment, our eDNA metabarcoding method revealed the presence of *L*. *alstonii* and *L*. *interrogans* in Okinawan environments for the first time. The low number of sequence reads that we obtained for some samples supports the low concentrations of these organisms in the environment, as previously reported^36^ (Fig. 3). Another reason for the low number of *Leptospira* reads obtained in our study is that the primers might not be perfect for our strategy and they might need to be improved. As expected, we found that leptospiral eDNA was correlated with rainfall on the sampling day (*r* = 0.8283–0.8692). Interestingly, even when we additionally considered the rainfall amount beginning 2 days before the sampling day, the correlation with leptospiral eDNA detection remained significant (*r* = 0.7488–0.8186), thus suggesting that the amount of rainfall is an environmental driver or factor favoring *Leptospira* survival and persistence outside the host.

The multiplex metabarcoding procedures that targeted bacterial universal and *Leptospira*-specific 16S rRNA gene regions revealed the association of *Leptospira* with 12 bacterial species. Although we can not prove that these species directly interact with *Leptospira*, our methodology might help to identify candidate species for further investigation. In addition, because the detection of 11 *Leptospira-*associated-species also correlated with rainfall amounts, our results suggest that the disturbance of river aggregates might increase the load of DNA from these microorganisms in the surrounding water. River aggregates are composed of bacteria, protozoa, and metazoans, and their stability depends on the availability of mucopolysaccharides, which essentially glue together organic and inorganic elements from aquatic and terrestrial environments^37^. A study that screened multiple paddy fields for the presence of environmental biofilm-forming organisms showed that diverse bacteria co-aggregated with *L*. *interrogans*^38^. In addition, *Sphingomonas*, which was identified in the previously cited study^38^, was likewise correlated with *Leptospira* in the current study (Fig. 4). Furthermore, the genus *Sphingomonas* supported leptospiral persistence and growth in an *in vitro* study^39^. *Chlamydiae*, which are obligate intracellular bacteria and have been described as symbionts of amoebae, also associated with *Leptospira*^40^. Collectively, our data suggest that the bacterial species that demonstrate an association with *Leptospira* might support their survival and persistence in the environment and that not only bacteria but other organisms, such as amoeba, might be present in environmental leptospiral aggregates.

Direct sequencing of eDNA has shown that most microbial lineages have not been isolated in pure culture. Earth microbiomes are highly diverse, and the reliable reads of 16S rRNA gene V4 regions that failed to yield Blast hits in our current study might represent as-yet-unidentified organisms^41^. Surprisingly, *lipL32* sequences occurred in several non-*Leptospira* species, and about half of the *lipL32* reads did not generate Blast hits (4,132 of 7,603). An *in silico* analysis identified orthologs of *Leptospira lipL32* in 10 marine bacteria and the spirochete *Treponema brennaborense*^42^. Data from our current study support the hypothesis that the distribution of *lipL32* orthologs in environmental ecosystems may be greater than previously estimated, and the horizontal transfer of DNA between diverse bacteria might accommodate multiple evolutionary mechanisms^43,44^.

Many mammalian species, including pinnipeds and bats^45,46^, as well as birds, amphibians, and reptiles, are known to carry pathogenic *Leptospira* species^47,48^. Wild ecosystems, such as those in northern Okinawa, are vastly biodiverse, and several animals may act as potential reservoirs of *Leptospira*. The assessment of animal hosts of pathogenic *Leptospira* has focused mainly on rodents and livestock animals because rodents play a major role in several risk factors established for *Leptospira* transmission such as poor sanitation and heavy rainfall, while leptospirosis in livestock animals has a negative economic influence due to reproductive problems^49–52^. Additional potential reservoir hosts need to be identified in endemic areas surrounding wild biomes. Moreover, the role of the rivers in the maintenance of infectious *Leptospira* needs to be analyzed since urban ornamental water fountains exposed to rodents and stray animals have been recently suggested to act as temporary carriers of pathogenic *Leptospira*^22^. Interestingly, our results showed that *Leptospira* species were associated with 10 vertebrate species in this region, thus identifying these animals as potential leptospiral hosts (Fig. 5). Among these species, boar (*Sus scrofa*) and fruit bat (*Pteropus dasymallus*) in particular can be considered potential *Leptospira* reservoirs. *Leptospira* in the kidneys of *S*. *scrofa* has been reported in Japan, the United States, and the Pantanal biome of Brazil^53–55^, whereas a growing number of studies have highlighted bats as *Leptospira* reservoir^56–58^. In contrast, our observed correlation of *Leptospira* with benthic fishes (*e*.*g*., eel, oriental weather loach, janitor fish, and Asian swamp eel) might reflect resuspension of sediment-borne eDNA into the river water due to increased flow during rainfall. Surprisingly, anti-leptospiral antibodies have been reported in 3 types of freshwater fishes (tilapia, catfish, and eels) in Tanzania^59^. *Leptospira* has not previously been reported in Okinawan river fishes, but the high correlation of leptospiral eDNA with eel and janitor fish (a kind of catfish; Fig. 5) that we disclosed might represent an interaction with *Leptospira* that warrants further investigation. *Cynops ensicauda* is only found in the Ryukyu Archipelago, which includes Okinawa Island^60^. This species is partially terrestrial, and adults frequently enter the water. Given that *L*. *interrogans* has been isolated from toads and frogs on the islands of Barbados^61^, amphibians may play a role in the leptospiral infection cycle^1^. The actual role of *C*. *ensicauda* as a leptospiral reservoir on Okinawa Island will be clarified in a future study.

Leptospirosis incidence is expected to increase in conjunction with global climate change and associated extreme weather events. The environmental factors associated with *Leptospira* species and leptospirosis transmission dynamics need to be investigated further. This study has several limitations since, the presence of eDNA is not always associated with infectious *Leptospira* and correlation of *Leptospira* with animals is not only influenced by bacterial/host relationship but by multiple factors not measurable by eDNA such as weather and changes in water flow. However, our results suggest that the DNA metabarcoding method we developed in this study is a powerful tool for better understanding the environmental phase of *Leptospira* and predicting human infection risks, as well as for identifying potential leptospiral reservoirs in a variety of environmental contexts. Furthermore, we showed that multiplex eDNA metabarcoding can be applied to detect diverse pathogenic organisms, their potential animal reservoirs and/or transmission vectors to define the infection cycles of human pathogens and to devise and implement control strategies for public health.

## Methods

### Environmental water sampling

Water sampling was conducted in 2 leptospirosis-endemic rivers (Okuma and Genka) in the northern region of the Okinawa Island, Japan. Considering that biofilms might be more common where the river flow is weaker, samples were taken close to the edge of the rivers. These rivers, surrounded by a wild environment, are recreational spots and numerous cases of leptospirosis have been reported after swimming in these rivers^6,7^. The water sampling was conducted four times during summer through early autumn (July to October) in 2017. The sampling sites comprised four locations (Fig. 1): one site (O1) at Okuma River and three sites (G1, G2, and G3) at Genka River. As a sampling unit, 500 mL of surface river water was manually collected by using a clean stainless steel ladle and stored in a disposable PET (polyethylene terephthalate) bottle. In each month, ten rounds of sampling were performed for each river, where a total of 5.0 L was collected at site O1, and 2.0, 1.5, and 1.5 L were taken at G1, G2, and G3, respectively. Temperature (°C), humidity (%), and water temperature (°C) at the sampling sites were recorded by using a portable thermometer and hygrometer. The sample bottles were immediately cooled on ice and brought to the laboratory (University of Ryukyus). Each unit of sample water (500 mL) was vacuum-filtered through a Whatman glass-fiber filter (pore size, 0.7 μm; diameter, 47 mm; Little Chalfont, Buckinghamshire, United Kingdom) and stored at −80 °C until DNA extraction. In preliminary experiments, we used cultured *L*. *interrogans* and PCR amplification and sequencing of the 16SrRNA gene to confirm the ability of Whatman glass-fiber filters to retain leptospiral cells (Supplementary Fig. S11).

### DNA extraction

Total eDNA was extracted from glass-fiber filters by using the DNeasy Blood and Tissue Kit (Qiagen, Hilden, Germany) according to a modified protocol. First, the filter was tightly packed into a spin column (Fast Gene Gel/PCR Extraction Kit, Nippon Genetics, Toyama, Japan) and centrifuged at 6,000 × *g* for 1 min to remove any water remaining. Then the column was moved into a new 1.5-mL tube and underwent proteinase K proteolysis (mixture of 10 μL of proteinase K, 90 μL of Buffer AL, and 200 μL of RNase-free water; Thermo Fisher Scientific, Waltham, MA, USA) at 56 °C for 15 min. The column then was centrifuged at 6,000 × *g* for 1 min to collect the lysate. To increase DNA yields, an additional 200 μL of TE buffer was poured into the filter, and the column was centrifuged again under the same conditions. Finally, 100 μL of Buffer AL (Thermo Fisher Scientific) and 300 μL of ethanol (Wako Pure Chemical, Japan) were added to the lysate. The obtained solution (ca. 900 μL) underwent subsequent steps of the manufacturer’s protocol for the DNeasy Kit using DNeasy Mini Spin Column (Qiagen). The extracted eDNA was eluted in 50 μL of RNase-free water and stored at −30 °C after verification of the DNA concentration and quality (OD_260/280_ > 1.80; Nanodrop 2000c Spectrophotometer, Thermo Fisher Scientific).

### PCR amplification for metabarcoding sequencing of *Leptospira* and other bacteria

To assess the *Leptospira* and bacterial microbiota in the river water samples, we used a two-step, tailed PCR method to amplify a portion of their 16S rRNA genes and of the *lipL32* gene of *Leptospira* from the prepared eDNA. In the first step, tailed primers for the targeted region of *Leptospira* 16S rRNA^21^, *lipL32*^32^, and universal bacterial 16S rRNA genes^15^ were generated by adding MiSeq sequencing priming sites and random hexamer nucleotides^10,15^ before using the primers in amplification reactions; addition of the random hexamers improves the base-call calibration of the MiSeq platform (Illumina, San Diego, CA, USA). Typically the target region of leptospiral 16S rRNA, *lipL32*, and bacterial 16S rRNA V4 was 330, 242, and 259 base pairs (bps), respectively, in length. These 3 PCR assays were performed in a multiplex manner by using Multiplex PCR Assay Kit version 2 (Takara, Shiga, Japan), in which the final concentration of each primer pair was set to 0.25 μM, 2.0 μL of template eDNA was used, and the total reaction volume was to 10.0 μL. The PCR conditions were as follows: 94 °C for 1 min followed by 35 cycles at 94 °C for 30 sec, 50 °C for 1 min, and 72 °C for 1 min.

For the second step, the first-round of PCR products were diluted 50-fold in RNase-free water. Dual-index tag sequences (D5, D7, A5, and A7 series; Illumina) and MiSeq flowcell binding sites were added to the first-round products by using Ex Taq Hot Start (Takara) as described previously^15^ (Fig. 2).

### PCR amplification for metabarcoding sequencing of vertebrates

We addressed the co-occurring, potential host organisms of *Leptospira* through eDNA metabarcoding analysis of vertebrate animals by using MiFish primers^10^. The typical 169-bp fragment of vertebrate mitochondrial DNA 12 S rRNA was amplified through two-step, tailed PCR analysis. In the first step, 2 PCR enzyme systems were applied: HiFi HotStart ReadyMix (KAPA Biosystems, Wilmington, MA USA) at an annealing temperature of 60 °C and PrimeSTAR HS DNA polymerase (Takara) with at 50 °C for annealing. In each system, the final concentration of MiFish-U primers was set to 0.30 μM with 2.0 μL of template eDNA. The PCR conditions were as follows: 95 °C for 3 min followed by 35 cycles at 98 °C for 20 sec, 60 °C for 15 sec, and 72 °C for 15 sec for the KAPA HiFi system and 94 °C for 3 min followed by 35 cycles at 98 °C for 10 sec, 50 °C for 15 sec, and 72 °C for 30 sec for the PrimeSTAR HS system. In the second step, the first-round PCR products were diluted 10-fold with RNase-free water, and dual-index tag sequences and flowcell binding sites were added by using Ex Taq HS (Takara) as described earlier^15^.

### Multiplex parallel DNA sequencing of metabarcoding PCR products

The tag-indexed second-round PCR products were pooled and underwent multiplex sequencing using MiSeq with V2 chemistry (Illumina). All second-round PCR products with unique combinations of dual-index tags were pooled in equal amounts for semi-quantitative sequencing and purified by using a MinElute Gel Extraction Kit (Qiagen) after size selection through 1.5% L03 agarose gel (Takara) electrophoresis. The eluted DNA solution was purified over a 1.8-fold amount (vol/vol) of AMPure XP beads (Agencourt Beckman Coulter, High Wycombe, Buckinghamshire, United Kingdom) according to a standard purification protocol using 70% ethanol. The obtained sequencing library was quantified by using a Qubit 2.0 fluorometer and dsDNA HS Assay Kit (Thermo Fisher Scientific), and the 20-pM library underwent 250 bp paired-end sequencing by using MiSeq Reagent Kit V2 (Illumina). The volume molarity was calculated according to the DNA concentration, average typical size of the PCR products, and molecular weight of a nucleotide (ca. 660 g per 1 bp).

### Metabarcoding sequencing data analysis

The raw sequence data generated through MiSeq analysis underwent primary processing according to sequence data quality. Total data quality was checked by using FastQC (http://www.bioinformatics.babraham.ac.uk/projects/fastqc/), and the low-quality (<10^−1^ error rate) 3′-tail of each sequence was removed by using DynamicTrim^62^. The tail-trimmed, paired-end sequences were merged by using FLASH^63^ and filtered by using custom Perl scripts to remove erroneous sequences containing basecall errors and those atypical in length compared with target fragments described earlier. Primer sequences were removed by using TagCleaner^64^, with a maximum four-base mismatch. Finally, redundant sequences of each sample were merged with keeping the count information by using UCLUST (derep_fulllength command)^65^.

The quality-filtered sequences underwent similarity-based taxonomic assignments by using the Blast plus program^66^ and the following databases: the NCBI nucleotide database^33^ for leptospiral 16S rRNA and *lipL32* genes; GreenGenes database^34^ for the universal bacterial 16S rRNA gene V4 region; and MitoFish^12,67^ and the NCBI nucleotide database for vertebrate mitochondrial 12S rRNA genes. For analysis of the leptospiral 16S rRNA and *lipL32* genes, species annotation was first performed at sequence similarity and *e*-value thresholds of 90% and 10^−3^, respectively. Then the annotations were confirmed or corrected according to molecular phylogenetic analysis with known reference sequences of representative *Leptospira* species (GenBank accession numbers and resulting phylogenetic trees are shown in Supplementary Figs S2 and S4). Multiple sequence alignments were performed by using Mafft^68^, and maximum-likelihood phylogenetic trees were estimated by using MEGA version 7.0.14^69^, which is based on a general time-reversible model of nucleotide substitution model^70^ with invariable sites and gamma correction.

For analysis of the universal bacterial 16S rRNA gene V4 region, reads sequenced more than 10 times in at least one sample were treated as representative sequences to eliminate potential PCR chimeras and sequencing errors. The sequence similarity and *e*-value thresholds of the species annotation were set to the same values as those for *Leptospira* sequences, described earlier.

For analysis of vertebrate mitochondrial 12 S rRNA genes, singleton sequences were re-mapped onto the remaining sequences (read number, ≥2) at 99% sequence similarity. The number of mapped singletons was incorporated into the count information of the subject sequence, and unmapped singletons were discarded. The Blast-based species annotation was conducted at increased sequence similarity and *e*-value cut-off threshold values of 97% and 10^−5^, respectively, because we considered that the database completeness of vertebrate mitochondrial DNA was higher than that for bacteria. Correlation and multivariate analyses were performed by using the software programs PAST^71^ and R (http://www.r-project.org/). Pearson’s product moment correlation coefficient (*r*) was applied for the analysis between sequence read numbers of *Leptospira* and bacteria or vertebrates. The two-sided significance level of 0.05 was adapted in this study, and corrected false discovery rates were estimated based on the Benjamini–Hochberg method for multiple testing. For the analysis of *Leptospira* and environmental parameters, both Pearson’s correlation coefficient (*r*) and Spearman’s rank correlation coefficient (*rho*) were examined, particularly using the 1/0/−1 coding data for weather (sunny, cloudy, or rainy; Supplementary Table S2). The data regarding rainfall amount (mm) during 2017 was taken from the website of the Japan Meteorological Agency (http://www.data.jma.go.jp/obd/stats/etrn/index.php) and weather (sunny, cloudy, or rainy) was recorded at the sampling site. The Nago City region was used to represent the Genka River, and Kunigami Village represented Okuma River. Partial correlations were calculated by using the R script pcor.R^72^.

## Supplementary information

Dataset 1

## Data Availability

Raw reads generated during the current study are available in the DDBJ Sequence Read Archive (DRA) under the accession numbers DRA007584 and DRA007585.
