# Supplementary material for: Environmental DNA metabarcoding to detect pathogenic Leptospira and associated organisms in leptospirosis-endemic areas of Japan
Source: Sci Rep. 2019 Apr 25;9:6575. doi: 10.1038/s41598-019-42978-1 (PMC6484013; doi:10.1038/s41598-019-42978-1)
Supplement: Supplementary file 1 — Dataset 1 [file 41598_2019_42978_MOESM1_ESM.pdf]

Supplementary Information

Environmental DNA metabarcoding to detect pathogenic *Leptospira* and associated organisms in leptospirosis-endemic areas of Japan

Yukuto Sato, Masaru Mizuyama, Megumi Sato, Toshifumi Minamoto, Ryosuke Kimura, Claudia Toma

Supplementary Table S1. Sample labels, locations and dates of sampling, and concentration and quality of the extracted DNA.

Sample name	Location [a]	Date of sampling and filtration	Date of DNA extraction	DNA concentration (ng/μL)	$OD_{260/280}$
O-11	O1	Jul. 10, 2017	Aug. 28, 2017	–1.00*	1.46
O-12	O1	Jul. 10, 2017	Aug. 28, 2017	–0.30*	0.47
O-13	O1	Jul. 10, 2017	Aug. 28, 2017	4.10	2.63
O-14	O1	Jul. 10, 2017	Aug. 28, 2017	1.30	1.98
O-15	O1	Jul. 10, 2017	Aug. 28, 2017	–0.70*	1.50
O-16	O1	Jul. 10, 2017	Aug. 28, 2017	–0.20*	–0.79
O-17	O1	Jul. 10, 2017	Aug. 28, 2017	0.30	3.34
O-18	O1	Jul. 10, 2017	Aug. 28, 2017	–0.60*	0.94
O-19	O1	Jul. 10, 2017	Aug. 28, 2017	–0.50*	2.00
O-20	O1	Jul. 10, 2017	Aug. 28, 2017	–1.20*	1.39
G-11	G1	Jul. 10, 2017	Aug. 28, 2017	1.80	3.82
G-12	G1	Jul. 10, 2017	Aug. 28, 2017	10.20	2.18
G-13	G1	Jul. 10, 2017	Aug. 28, 2017	1.20	10.32
G-14	G2	Jul. 10, 2017	Aug. 28, 2017	–1.80*	1.84
G-15	G2	Jul. 10, 2017	Aug. 28, 2017	2.40	1.64
G-16	G2	Jul. 10, 2017	Aug. 28, 2017	0.90	4.59
G-17	G3	Jul. 10, 2017	Aug. 28, 2017	0.60	–2.25
G-18	G3	Jul. 10, 2017	Aug. 28, 2017	12.90	2.35
G-19	G3	Jul. 10, 2017	Aug. 28, 2017	1.90	2.27
G-20	G3	Jul. 10, 2017	Aug. 28, 2017	1.80	3.64
O-21	O1	Aug. 07, 2017	Sep. 06, 2017	6.00	2.23
O-22	O1	Aug. 07, 2017	Sep. 06, 2017	7.80	2.25
O-23	O1	Aug. 07, 2017	Sep. 06, 2017	8.40	2.12
O-24	O1	Aug. 07, 2017	Sep. 06, 2017	6.60	2.28
O-25	O1	Aug. 07, 2017	Sep. 06, 2017	11.80	2.09
O-26	O1	Aug. 07, 2017	Sep. 06, 2017	9.80	1.93
O-27	O1	Aug. 07, 2017	Sep. 06, 2017	9.00	2.20
O-28	O1	Aug. 07, 2017	Sep. 06, 2017	4.00	3.65
O-29	O1	Aug. 07, 2017	Sep. 06, 2017	2.10	4.07
O-20	O1	Aug. 07, 2017	Sep. 06, 2017	2.90	2.34
G-21	G1	Aug. 07, 2017	Sep. 08, 2017	5.40	2.69
G-22	G1	Aug. 07, 2017	Sep. 08, 2017	9.20	2.08
G-23	G1	Aug. 07, 2017	Sep. 08, 2017	3.90	2.38

G-24	G2	Aug. 07, 2017	Sep. 08, 2017	4.90	2.04
G-25	G2	Aug. 07, 2017	Sep. 08, 2017	0.60	–2.94
G-26	G2	Aug. 07, 2017	Sep. 08, 2017	5.40	2.33
G-27	G3	Aug. 07, 2017	Sep. 08, 2017	7.40	2.07
G-28	G3	Aug. 07, 2017	Sep. 08, 2017	2.20	3.77
G-29	G3	Aug. 07, 2017	Sep. 08, 2017	2.70	2.86
G-30	G3	Aug. 07, 2017	Sep. 08, 2017	2.20	10.20
O-31	O1	Sep. 11, 2017	Oct. 17, 2017	3.40	2.02
O-32	O1	Sep. 11, 2017	Oct. 17, 2017	1.40	–1.84
O-33	O1	Sep. 11, 2017	Oct. 17, 2017	–0.30*	0.48
O-34	O1	Sep. 11, 2017	Oct. 17, 2017	2.40	12.68
O-35	O1	Sep. 11, 2017	Oct. 17, 2017	2.30	4.93
O-36	O1	Sep. 11, 2017	Oct. 17, 2017	6.30	2.21
O-37	O1	Sep. 11, 2017	Oct. 17, 2017	4.00	2.33
O-38	O1	Sep. 11, 2017	Oct. 17, 2017	1.70	–16.95
O-39	O1	Sep. 11, 2017	Oct. 17, 2017	1.70	–2.21
O-40	O1	Sep. 11, 2017	Oct. 17, 2017	1.80	–1.98
G-31	G1	Sep. 11, 2017	Oct. 17, 2017	0.40	–0.37
G-32	G1	Sep. 11, 2017	Oct. 17, 2017	3.80	3.88
G-33	G1	Sep. 11, 2017	Oct. 17, 2017	0.60	–0.89
G-34	G2	Sep. 11, 2017	Oct. 17, 2017	4.20	4.03
G-35	G2	Sep. 11, 2017	Oct. 17, 2017	0.50	–0.39
G-36	G2	Sep. 11, 2017	Oct. 17, 2017	1.20	–1.08
G-37	G3	Sep. 11, 2017	Oct. 17, 2017	3.20	3.56
G-38	G3	Sep. 11, 2017	Oct. 17, 2017	–0.30*	0.27
G-39	G3	Sep. 11, 2017	Oct. 17, 2017	3.60	2.80
G-40	G3	Sep. 11, 2017	Oct. 17, 2017	–0.30*	0.30
O-41	O1	Oct. 16, 2017	Oct. 23, 2017	2.40	3.83
O-42	O1	Oct. 16, 2017	Oct. 23, 2017	0.50	–1.31
O-43	O1	Oct. 16, 2017	Oct. 23, 2017	0.20	–0.14
O-44	O1	Oct. 16, 2017	Oct. 23, 2017	0.00	–0.04
O-45	O1	Oct. 16, 2017	Oct. 23, 2017	3.30	3.30
O-46	O1	Oct. 16, 2017	Oct. 23, 2017	13.10	2.00
O-47	O1	Oct. 16, 2017	Oct. 23, 2017	1.60	39.14
O-48	O1	Oct. 16, 2017	Oct. 23, 2017	0.80	–2.45
O-49	O1	Oct. 16, 2017	Oct. 23, 2017	5.40	2.20
O-50	O1	Oct. 16, 2017	Oct. 23, 2017	1.90	2.95
G-41	G1	Oct. 16, 2017	Oct. 23, 2017	2.10	6.15
G-42	G1	Oct. 16, 2017	Oct. 23, 2017	15.40	2.08
G-43	G1	Oct. 16, 2017	Oct. 23, 2017	2.00	–10.66
G-44	G2	Oct. 16, 2017	Oct. 23, 2017	9.00	2.77
G-45	G2	Oct. 16, 2017	Oct. 23, 2017	1.30	3.72
G-46	G2	Oct. 16, 2017	Oct. 23, 2017	5.80	2.38
G-47	G3	Oct. 16, 2017	Oct. 23, 2017	8.50	2.35
G-48	G3	Oct. 16, 2017	Oct. 23, 2017	3.40	2.86
G-49	G3	Oct. 16, 2017	Oct. 23, 2017	18.60	2.17
G-50	G3	Oct. 16, 2017	Oct. 23, 2017	7.60	2.93

[*] DNA concentration was below the lower limit of quantification (NanoDrop 2000c Spectrophotometer, Thermo Fisher Scientific, Waltham, MA, USA)

[a] Details regarding locations of the sampling sites are given in Fig. 1.

Supplementary Table S2. Environmental parameters at the time of sampling in each sampling site.

	G11−20 [a]	G21−30 [a]	G31−40 [a]	G41−50 [a]	O11−20	O21−30	O31−40	O41−50
Temperature (°C)	31.9	31.6	32.2	33	36.9	29.2	32.1	36
Humidity (%)	74.2	61.3	72	77.1	60.5	56.7	69	63.4
Water temperature (°C)	27.1	26.9	28.7	25.3	27.5	27.2	28.5	26.4
Weather [b]	1	0	1	0	1	0	1	1

[a] Location G (Genka River) series presents average scores for the 3 sampling sites.

[b] Weather: 1, sunny; 0, cloudy; −1, rainy.

Supplementary Table S3. Pearson's correlation coefficients between *Leptospira* and vertebrate eDNA detection (read numbers) or rainfall amount (mm).

	KP60 [a]		PS50 [b]	
	Lipl32	*Lepto–16S*	*Lipl32*	*Lepto–16S*
Rainfall_1day	0.8692	0.8283	0.8692	0.8283
Rainfall_3day	0.7488	0.8186	0.7488	0.8186
Lipl32	1.0000	0.8946	1.0000	0.8946
Lepto-16S	0.8946	1.0000	0.8946	1.0000
Acanthopagrus sivicolus	0.0000	0.0000	–0.2228	0.0221
Acanthurus dussumieri	0.0000	0.0000	–0.2909	–0.3166
Anguilla japonica	0.7013	0.6661	–0.2075	–0.2309
Anguilla luzonensis	–0.2909	–0.3166	0.9444	0.9153
Anguilla marmorata	0.3716	0.1635	0.6260	0.4545
Apogon doryssa	–0.2541	–0.2858	–0.2909	–0.3166
Awaous melanocephalus	–0.3021	0.0045	–0.3621	–0.1063
Bothus pantherinus	–0.2541	–0.2858	0.0000	0.0000
Canis lupus familiaris	–0.3723	–0.4142	–0.2228	0.0221
Canthigaster amboinensis	–0.0593	–0.2242	0.0000	0.0000
Caragobius urolepis	0.1313	–0.1319	0.0000	0.0000
Caranx sexfasciatus	–0.2228	0.0221	–0.2306	–0.1041
Carassius auratus auratus	0.1750	0.0740	–0.2572	–0.4643
Casmerodius albus	–0.2228	0.0221	–0.2228	0.0221
Chaetodon vagabundus	0.0000	0.0000	–0.2713	–0.2865
Chelon macrolepis	–0.2689	–0.3020	0.0837	0.0269
Choerodon schoenleinii	0.0000	0.0000	–0.1097	0.1761
Chrysiptera glauca	–0.2228	0.0221	0.0000	0.0000
Columba janthina janthina	0.0000	0.0000	–0.1390	–0.1549
Corvus splendens	0.0000	0.0000	0.5694	0.5799
Cynops ensicauda	0.3520	0.3222	0.9444	0.9153
Cyprinus carpio	0.0753	0.1399	–0.2528	–0.2831
Egretta garzetta	0.0000	0.0000	–0.2228	0.0221
Eleotris fusca	–0.3247	–0.0104	–0.4313	–0.2526
Gambusia affinis	0.1313	–0.1319	–0.2909	–0.3166
Glossogobius celebius	–0.1097	0.1761	0.0000	0.0000
Herpestes javanicus	0.0000	0.0000	–0.1390	–0.1549
Homo sapiens	–0.1170	–0.1276	0.1128	0.0451
Hypsipetes amaurotis	0.0000	0.0000	–0.1390	–0.1549
Kuhlia marginata	–0.3825	–0.0004	–0.4756	–0.2436
Kuhlia rupestris	–0.4076	–0.0457	–0.3729	–0.2478
Misgurnus anguillicaudatus	0.0000	0.0000	0.9444	0.9153
Monopterus albus	0.0000	0.0000	0.7311	0.4047
Mugil cephalus	–0.4303	–0.1144	–0.2761	–0.1758
Mulloidichthys vanicolensis	–0.2909	–0.3166	0.0000	0.0000
Mus musculus domesticus	–0.2080	–0.2644	–0.2541	–0.2858
Nematalosa come	–0.2541	–0.2858	0.0000	0.0000
Neoniphon sammara	0.0000	0.0000	–0.2909	–0.3166

Oncorhynchus mykiss	0.1313	–0.1319	0.0000	0.0000
Oncorhynchus nerka	0.1313	–0.1319	0.0000	0.0000
Ophieleotris sp. Tametomohaze	–0.1390	–0.1549	0.0000	0.0000
Oreochromis aureus	0.0000	0.0000	–0.2541	–0.2858
Oreochromis niloticus	0.1392	–0.1047	–0.2700	–0.2968
Oreochromis sp. red tilapia	–0.2046	–0.3288	0.0000	0.0000
Ostorhinchus cookii	0.0000	0.0000	–0.2541	–0.2858
Ostorhinchus doederleini	–0.2541	–0.2858	0.0000	0.0000
Ostracion immaculatus	0.0000	0.0000	–0.2909	–0.3166
Parupeneus ciliatus	0.0000	0.0000	0.1313	–0.1319
Pelodiscus sinensis	–0.1743	0.0840	–0.2463	–0.2457
Pempheris xanthoptera	–0.2541	–0.2858	–0.2541	–0.2858
Plecoglossus altivelis	–0.1390	–0.1549	–0.1390	–0.1549
Poecilia reticulata	–0.3009	0.0467	–0.2977	–0.0101
Pteropus dasymallus	–0.1390	–0.1549	0.8615	0.8937
Pterygoplichthys disjunctivus	0.0000	0.0000	0.9444	0.9153
Rastrelliger kanagurta	–0.2909	–0.3166	0.0000	0.0000
Rattus tanezumi	–0.2228	0.0221	–0.3301	–0.2741
Redigobius bikolanus	–0.2228	0.0221	–0.2228	0.0221
Rhinogobius brunneus Okinawa	–0.3602	–0.0136	–0.1174	–0.0468
Rhinogobius giurinus	–0.3511	0.0379	–0.3496	–0.1751
Rhinogobius sp CBM-ZF12303	–0.3451	0.0105	–0.3055	–0.2128
Rhyacichthys aspro	–0.1390	–0.1549	0.0000	0.0000
Sargocentron ittodai	0.0000	0.0000	–0.2909	–0.3166
Scatophagus argus	0.0000	0.0000	–0.0340	–0.2879
Schismatogobius roxasi	–0.1097	0.1761	–0.1097	0.1761
Sicyopterus japonicus	–0.1797	–0.1443	–0.1485	–0.1495
Sicyopterus lagocephalus	–0.1389	–0.1541	–0.1384	–0.1538
Siganus guttatus	–0.2541	–0.2858	0.1313	–0.1319
Stenogobius sp. Tanekawahaze	–0.2358	0.1676	–0.2228	0.0221
Stiphodon alcedo	0.0000	0.0000	–0.2228	0.0221
Stiphodon percnopterygionus	–0.2863	0.1154	–0.3560	–0.1652
Sus scrofa breed type I Lanyu	0.9444	0.9153	0.9444	0.9153
Trachurus japonicus	0.0000	0.0000	–0.2909	–0.3166
Tridentiger kuroiwae	–0.1723	0.1436	0.2161	0.2733
Zosterops erythropleurus	0.0000	0.0000	–0.2228	0.0221

[a] KP60 denotes results from PCR amplification using KAPA HiFi HotStart ReadyMix kit (KAPA Biosystems, Wilmington, MA, USA) at an annealing temperature of 60 °C.

[b] PS50 denotes results from the PCR using PrimeSTAR HS DNA polymerase (Takara, Shiga, Japan) at an annealing temperature of 50 °C.

Supplementary Figures

Supplementary Figure S1. Photos of river water sampling at Genka and Okuma Rivers in October 2017.

Supplementary Figure S2. Molecular phylogenetic tree for leptospiral 16S rRNA genes. In total 282 nucleotide sites from environmental DNA sequences determined by the present study (shown in gray shading) were aligned and analyzed with known reference sequences of representative species of *Leptospira* (GenBank accession numbers are shown within sequence names).

Supplementary Figure S3. Environmental detection of *Leptospira lipL32* gene. The numbers of sequence reads detected in each sample are shown as colored matrices in orange shading. Orange bars indicate the total number of *lipL32* reads per sample summed across *Leptospira* species. Blue bars indicate the amount of rainfall (mm) on the sampling day (left column) and that including the 2 days before sampling, the day of sampling, and the day afterward (right column). O1, G1, G2, and G3 indicate sampling locations Okuma-1 and Genka-1, -2, and -3, respectively. G-11−G-50 and O-11−O-50 denote sample names.

Supplementary Figure S4. Molecular phylogenetic tree for *Leptospira lipL32* gene. In total 158 nucleotide sites from environmental DNA sequences determined by the present study (shown in gray shading; primary Blast-based species annotations are described with sequence names) were aligned and analyzed with known reference sequences of representative pathogenic species of *Leptospira* (GenBank accession numbers are shown with sequence names).

Supplementary Figure S5. Heatmap representation of relative sequence read counts of bacterial species detected by matabarcoding analysis of 16S rRNA gene V4 region. Columns indicate detected bacterial species, and rows indicate river water samples; O1, G1, G2, and G3 denote sampling locations Okuma-1 and Genka-1, -2, and -3, respectively. G-11−G-50 and O-11−O-50 indicate sample names. Pink-colored matrices indicate the relative sequence read counts standardized to the logarithm of

counts per million (cpm) reads within each sample.

Supplementary Figure S6. Bacteriome profiles and their temporal changes in Genka and Okuma Rivers. Ordinations of two-dimensional nonmetric multidimensional scaling of the standardized profiles of bacteriomes were estimated by using the 16S rRNA gene V4 region data for each sample. G-11−G-50 and O-11−O-50 indicate sample names. Horizontal and vertical axes correspond to the estimated two-dimensional coordinate 1 and 2 where ranked differences in similarity scores based on Pearson's correlation coefficients (*r*) were preserved. A normalized stress value of this plot was 0.207, and the determination factor R^2 values of coordinate 1 and 2 were 0.579 and 0.203, respectively.

Supplementary Figure S7. Heatmap representation of relative sequence read counts of vertebrate species detected by matabarcoding analysis of the mitochondrial 12S rRNA gene. Columns indicate detected species, and rows indicate river water samples; O1, G1, G2, and G3 denote the sampling locations Okuma-1 and Genka-1, -2, and -3, respectively. G-11−G-50 and O-11−O-50 indicate sample names. Pink-colored matrices indicate relative sequence read counts standardized to logarithm of counts per million (cpm) reads within each sample.

Supplementary Figure S8. Correlation coefficients between the 18 bacteria species correlated with *Leptospira* detection and 74 vertebrates detected in this study. Columns denote the numbers of operational taxonomic units (OTUs) of the bacterial species shown in Fig. 4C. Gray shading indicates OTUs that were significantly correlated with leptospiral 16S rRNA gene detection ($P < 0.05$), and asterisks show that their partial correlation corrected for rainfall was significant ($P < 0.05$). Rows denote vertebrate species. Pink-colored matrices indicate positive correlation, whereas blue shading indicates negative correlation. Yellow shading denotes the 10 vertebrate species that were highly correlated with *Leptospira* detection (indicated by the black bar in Fig. 5).

Supplementary Figure S9. Partial correlation coefficients between 18 bacteria species and 74 vertebrates after correction for *Leptospira* detection. Columns denote

the numbers of operational taxonomic unit (OTUs) of the bacterial species highlighted in Fig. 4C. Gray shading indicates OTUs that were significantly correlated with leptospiral 16S rRNA gene detection ($P < 0.05$), and asterisks show that their partial correlation after correction for rainfall remained significant ($P < 0.05$). Rows denote vertebrate species. Matrices colored in pink indicate positive correlations, whereas blue shading shows negative correlations. Yellow shading denotes the 10 vertebrate species that were highly correlated with *Leptospira* detection (indicated by the black bar in Fig. 5).

Supplementary Figure S10. Statistical significance of the partial correlation coefficients between 18 bacteria species and 74 vertebrates after correction for *Leptospira* detection. Columns denote the numbers of operational taxonomic units (OTUs) of the bacterial species highlighted in Fig. 4C. Gray shading indicates OTUs that were significantly correlated with leptospiral 16S rRNA gene detection ($P < 0.05$), and asterisks show that their partial correlation after correction for rainfall remained significant ($P < 0.05$). Rows denote vertebrate species. Green shading indicates the level of statistical significance, and yellow shading denotes the 10 vertebrate species that were highly correlated with *Leptospira* detection (indicated by the black bar in Fig. 5).

Supplementary Figure S11. Preliminary experiment for collections of leptospiral cells by Whatman glass-fiber filters. (A) Bottles of the cultured *Leptospira interrogans* diluted to concentrations of 10^3, 10^4, 10^5, 10^6, and 4 x 10^6 cells per milliliter by ultrapure water obtained by a Milli-Q Water System (Millipore Corporation, Bedford, MA). Five litters of these solutions were vacuum-filtered through by Whatman glass-fiber filter (pore size, 0.7 µm; diameter, 47 mm; Little Chalfont, Buckinghamshire, United Kingdom). Half of the filters was respectively subjected to the DNA extraction by using DNeasy Blood and Tissue Kit or PowerSoil Kit (Qiagen, Hilden, Germany). By the former kit, 0.172, 0.257, 2.850, 17.00, and 64.20 ng/µL of total DNA was extracted from the five bottles of the above order, respectively. By the latter kit, 0.053, 0.000, 0.235, 0.843, and 0.336 ng/µL of DNA were obtained, respectively. (B) PCR amplification of bacterial 16S rRNA V4 region. Ten DNA samples obtained above were subjected to PCR experiments and sequencing by Illumina

MiSeq platform (Illumina, San Diego, CA, USA) according to the method described in Sato *et al*. (2015)[1]. Nega denotes negative control sample (ultrapure water) and M denotes 100 bp DNA Ladder H3 RTU (Nippon Genetics, Toyama, Japan). Target sequences (378 bp) were successfully amplified. (C) A typical sequence accounts for the majority of DNA sequences obtained from the above samples. We confirmed that ≥ 97.0% of these sequences were from *L. interrogans* based on the nucleotide Blast analysis (https://blast.ncbi.nlm.nih.gov/Blast.cgi). A few percent of them were estimated to be derived from bacteria in tap water according to the same analysis.

References

[1] Sato, Y. *et al*. Inter-individual differences in the oral bacteriome are greater than intra-day fluctuations in individuals. *PLoS One* **10**, e0131607 (2015).

Genka River (G-46) sampling

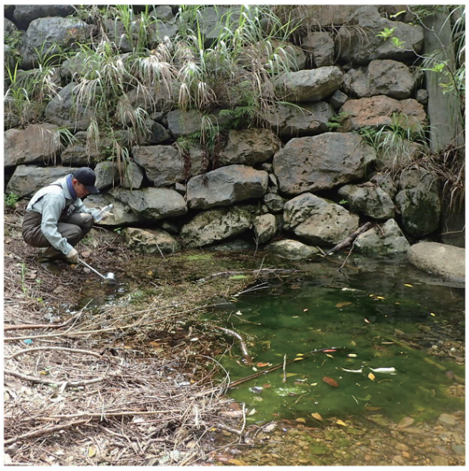

Genka River (G-50) sampling

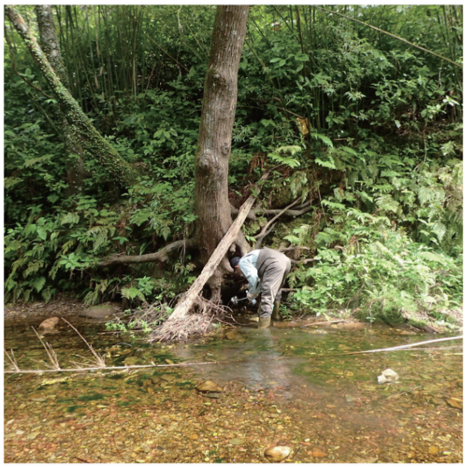

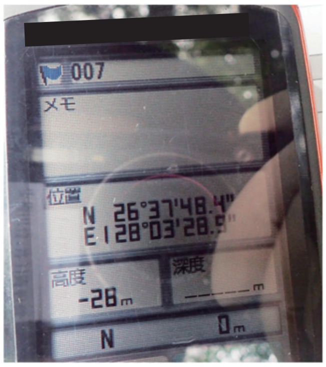

Genka River G41 GPS

Okuma River (O-45) sampling

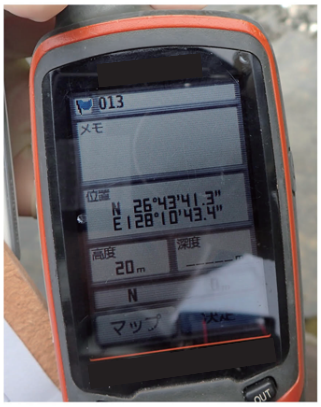

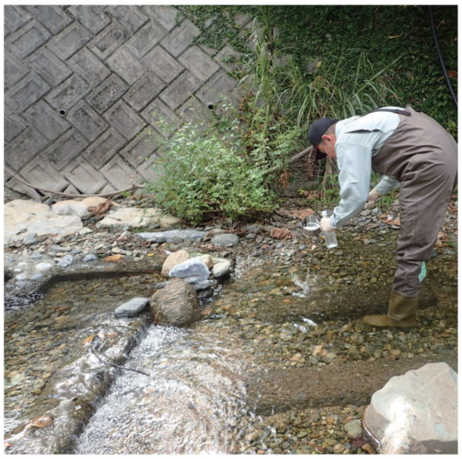

Supplementary Figure S1

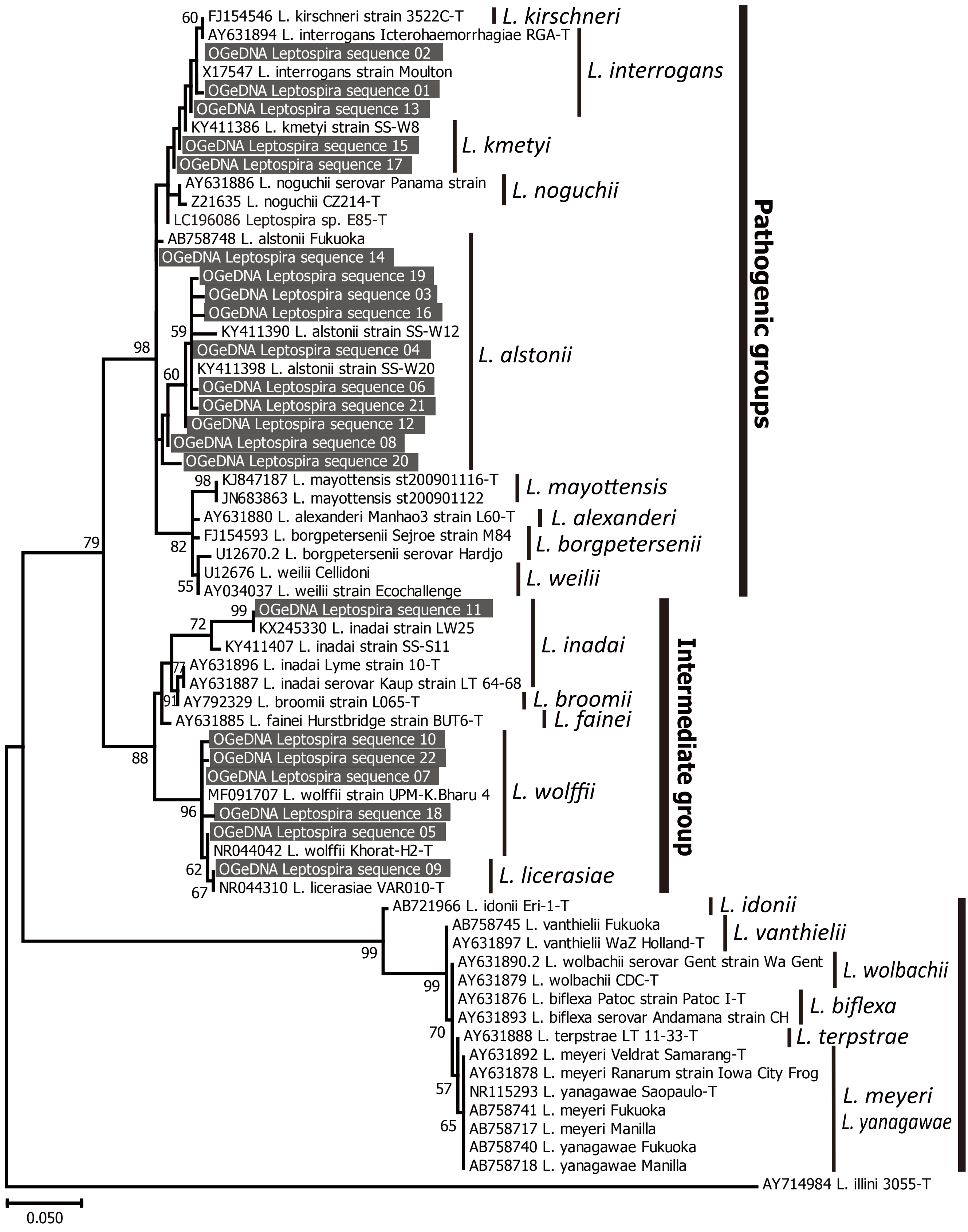

Supplementary Figure S2

River	Month	Site	Sample	*Leptospira*: *L. alstonii*	*Leptospira*: *Leptospira* sp. S654/671	*Leptospira*: *Leptospira* sp.	Other bacteria: *Bacillus cytotoxicus*	Other bacteria: *Bartonella* sp. JB63	Other bacteria: *Cupriavidus* sp. USMAA1020	Other bacteria: *Curvibacter putative*	Other bacteria: *Gemmatimonas phototrophica*	Other bacteria: *Gloeobacter violaceus*	Other bacteria: *Lysinibacillus sphaericus*	Other bacteria: *Pantoea vagans*	Total *Leptospira* reads	Rainfall (mm): Sampling day	Rainfall (mm): Three days total
Genka River	Jul.	G1	G-11	9	0	0	0	0	0	0	0	0	0	0	9	0	3
			G-12	0	0	0	0	0	0	0	0	0	0	0	0	0	3
			G-13	0	0	0	0	0	0	0	0	0	0	0	0	0	3
		G2	G-14	0	0	0	0	0	0	0	0	0	0	0	0	0	3
			G-15	1	0	0	0	0	0	0	0	0	0	0	1	0	3
			G-16	1	0	0	0	0	0	0	0	0	0	0	1	0	3
		G3	G-17	0	0	0	0	0	0	0	0	0	0	0	0	0	3
			G-18	0	30	0	0	0	0	0	0	0	0	0	30	0	3
			G-19	0	0	0	0	0	0	0	0	0	0	0	0	0	3
			G-20	12	1	0	0	0	0	0	0	0	4	0	13	0	3
	Aug.	G1	G-21	0	0	0	0	0	0	0	0	0	0	0	0	17	21
			G-22	0	0	0	0	0	0	0	0	0	0	0	0	17	21
			G-23	56	79	0	0	0	0	0	2	0	0	0	135	17	21
		G2	G-24	1		0	0	0	0	0	0	0	0	0	1	17	21
			G-25	0	0	0	0	0	0	0	0	0	0	0	0	17	21
			G-26	40	0	0	0	0	0	0	0	0	0	0	40	17	21
		G3	G-27	46	0	0	0	0	0	0	0	0	0	0	46	17	21
			G-28	0	0	0	0	0	0	0	0	0	0	0	0	17	21
			G-29	1	0	0	0	0	0	0	0	0	0	0	1	17	21
			G-30	0	0	0	0	0	0	0	0	0	0	0	0	17	21
	Sep.	G1	G-31	98	0	0	0	0	0	0	0	0	0	0	98	0	27.5
			G-32	125	0	0	0	0	0	0	0	0	0	0	125	0	27.5
			G-33	4	0	0	0	0	0	2	0	0	0	0	4	0	27.5
		G2	G-34	0	0	0	0	0	0	0	0	0	0	0	0	0	27.5
			G-35	0	0	0	0	0	0	0	0	0	0	0	0	0	27.5
			G-36	0	0	0	0	0	0	0	0	0	0	0	0	0	27.5
		G3	G-37	37	0	0	0	0	0	0	0	0	0	0	37	0	27.5
			G-38	2	0	0	0	0	0	0	0	0	0	0	2	0	27.5
			G-39	0	0	0	0	0	0	0	0	0	0	0	0	0	27.5
			G-40	0	0	0	0	0	0	0	0	0	0	0	0	0	27.5
	Oct.	G1	G-41	0	0	0	0	0	0	0	0	0	0	0	0	0	4.5
			G-42	62	0	0	0	0	0	0	0	0	0	0	62	0	4.5
			G-43	38	0	0	0	2	0	0	0	0	0	0	38	0	4.5
		G2	G-44	0	0	0	0	0	0	0	0	0	0	0	0	0	4.5
			G-45	0	0	0	0	0	0	0	0	0	0	0	0	0	4.5
			G-46	0	0	0	18	0	0	0	0	0	0	0	0	0	4.5
		G3	G-47	0	0	0	0	0	0	0	0	0	0	0	0	0	4.5
			G-48	0	0	0	0	0	0	0	0	0	0	0	0	0	4.5
			G-49	0	0	0	0	0	0	0	0	0	0	0	0	0	4.5
			G-50	0	0	0	0	0	0	0	0	0	0	0	0	0	4.5
															0		
Okuma River	Jul.	O1	O-11	0	0	0	0	0	0	0	0	0	0	0	0	0	1.5
			O-12	0	0	0	0	0	0	0	0	0	0	0	0	0	1.5
			O-13	0	0	0	0	0	0	0	0	0	0	0	0	0	1.5
			O-14	0	0	0	0	0	0	0	0	0	0	4	0	0	1.5
			O-15	0	0	0	0	0	0	0	0	0	0	0	0	0	1.5
			O-16	0	0	0	0	0	0	0	0	0	0	0	0	0	1.5
			O-17	0	0	0	0	0	0	0	0	0	0	0	0	0	1.5
			O-18	0	0	0	0	0	0	0	0	0	0	0	0	0	1.5
			O-19	0	0	0	0	0	0	0	0	0	0	0	0	0	1.5
			O-20	0	0	0	0	0	0	0	0	0	0	0	0	0	1.5
	Aug.	O1	O-21	269	0	0	0	0	0	0	0	0	0	0	269	41.5	45
			O-22	0	100	0	0	0	0	0	0	3	0	0	100	41.5	45
			O-23	280	0	0	0	0	0	0	1	0	0	0	280	41.5	45
			O-24	100	0	1	0	0	4	0	0	0	0	0	101	41.5	45
			O-25	644	0	1	0	0	0	0	0	0	0	0	645	41.5	45
			O-26	164	54	0	0	0	0	0	0	0	0	0	218	41.5	45
			O-27	176	0	0	0	0	0	0	0	0	0	0	176	41.5	45
			O-28	0	0	0	0	0	0	0	0	0	0	0	0	41.5	45
			O-29	1	0	0	0	0	0	0	0	0	0	0	1	41.5	45
			O-30	24	0	0	0	0	0	0	0	0	0	0	24	41.5	45
	Sep.	O1	O-31	0	0	0	0	0	0	0	0	0	0	0	0	0	2
			O-32	27	0	0	0	0	0	0	0	0	0	0	27	0	2
			O-33	0	0	0	0	0	0	0	0	0	0	0	0	0	2
			O-34	0	0	0	0	0	0	0	0	0	0	0	0	0	2
			O-35	168	0	0	0	0	0	0	0	0	0	0	168	0	2
			O-36	321	0	0	0	0	0	0	0	0	0	0	321	0	2
			O-37	41	0	0	0	0	0	0	0	0	0	0	41	0	2
			O-38	1	0	0	0	0	0	0	0	0	0	0	1	0	2
			O-39	62	0	0	0	0	0	0	0	0	0	0	62	0	2
			O-40	0	0	0	0	0	0	0	0	0	0	0	0	0	2
	Oct.	O1	O-41	0	0	0	0	0	0	0	0	0	0	0	0	1.5	21.5
			O-42	0	0	0	0	0	0	0	0	0	0	0	0	1.5	21.5
			O-43	92	0	0	0	0	0	0	0	0	0	0	92	1.5	21.5
			O-44	0	0	0	0	0	0	0	0	0	0	0	0	1.5	21.5
			O-45	191	0	0	0	0	0	0	0	0	0	0	191	1.5	21.5
			O-46	0	1	0	0	0	0	0	0	0	0	0	1	1.5	21.5
			O-47	3	0	0	0	0	0	0	0	0	0	0	3	1.5	21.5
			O-48	1	0	0	0	0	0	0	0	0	0	0	1	1.5	21.5
			O-49	22	0	0	0	0	0	0	0	0	0	0	22	1.5	21.5
			O-50	30	0	0	0	0	0	0	0	0	0	0	30	1.5	21.5
			Nega01	0	0	0	0	0	0	0	0	0	0	0			
			Nega02	0	0	0	0	0	0	0	0	0	0	0			

Supplementary Figure S3

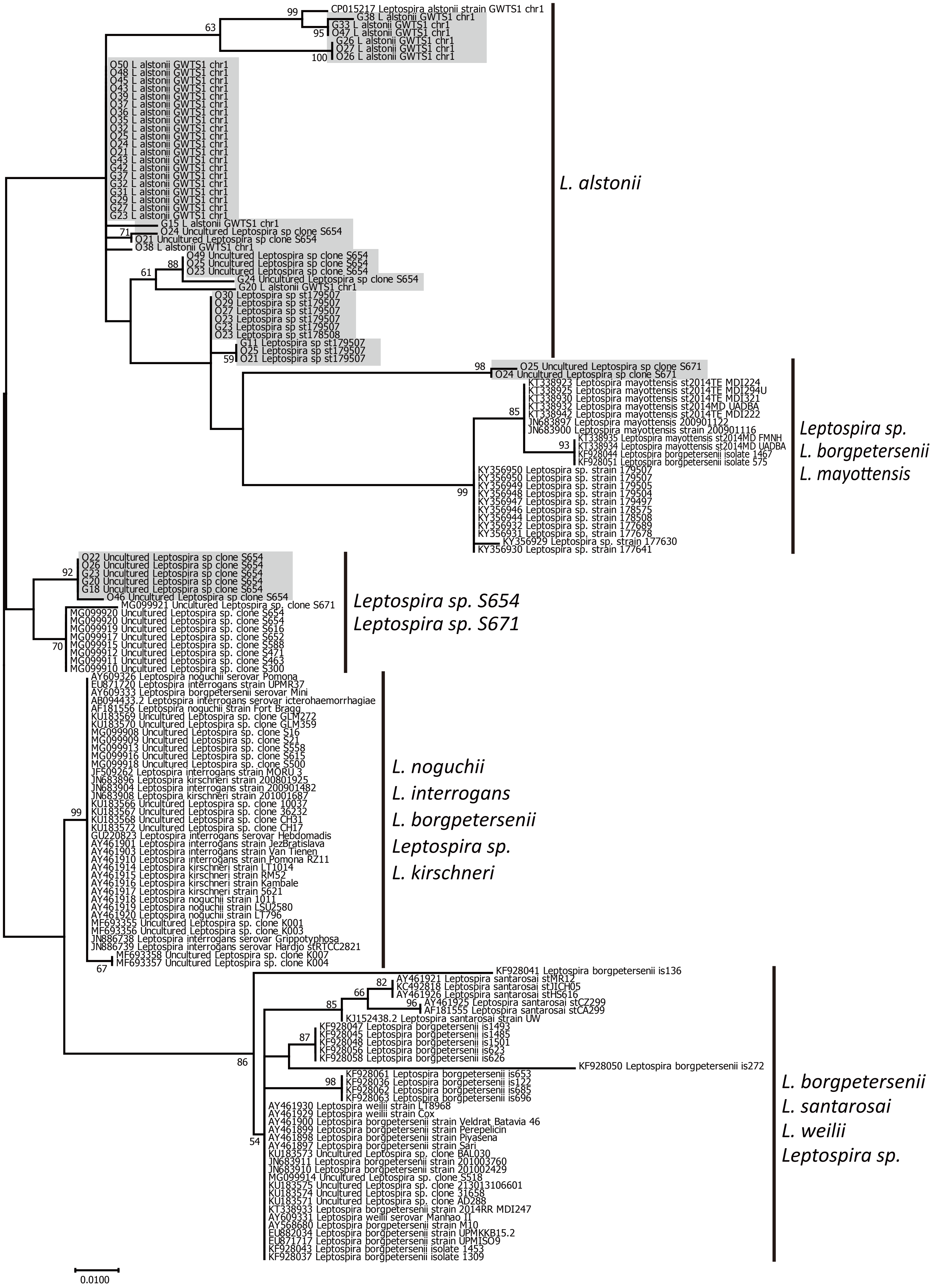

Supplementary Figure S4

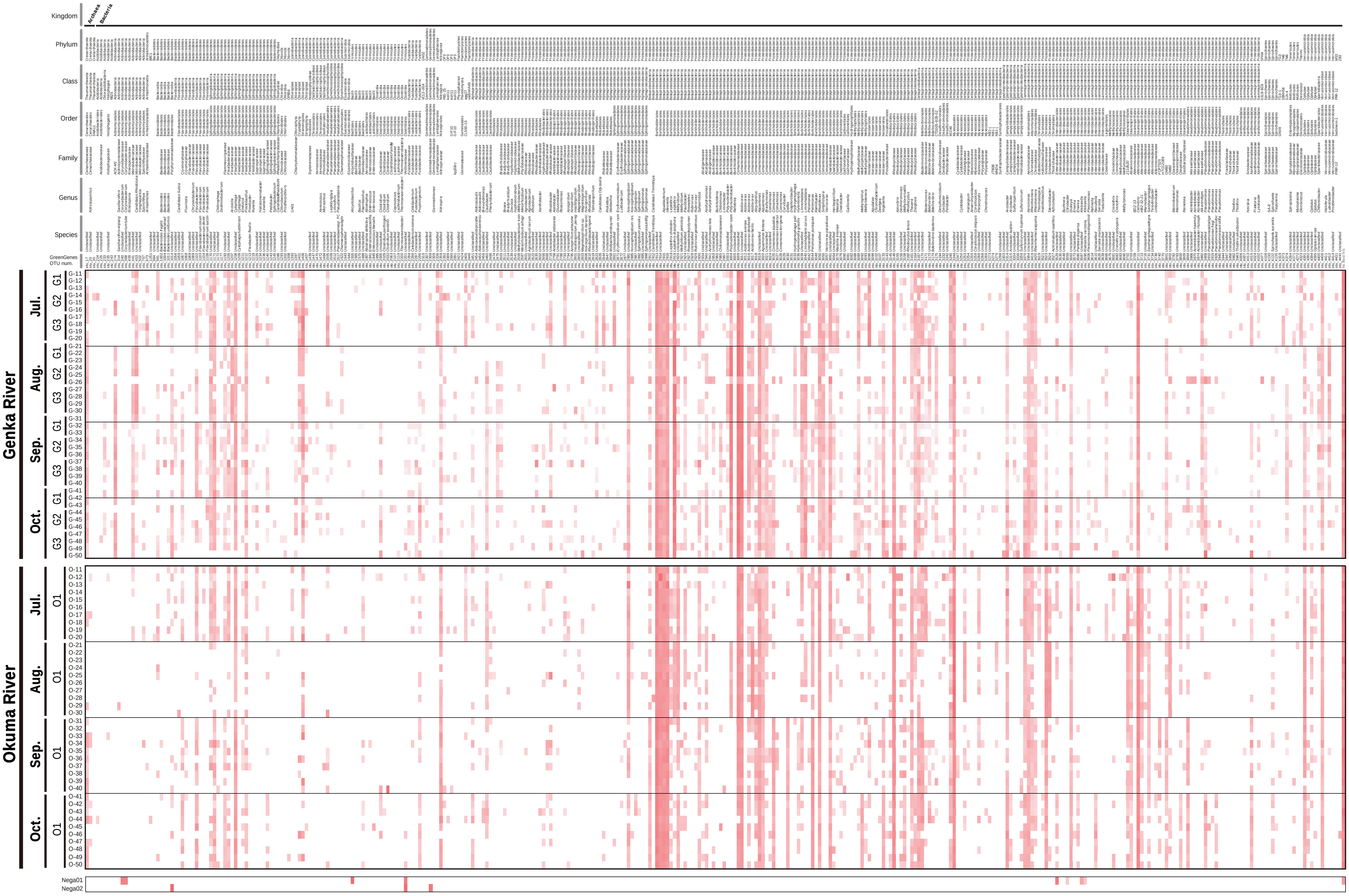

Supplementary Figure S5

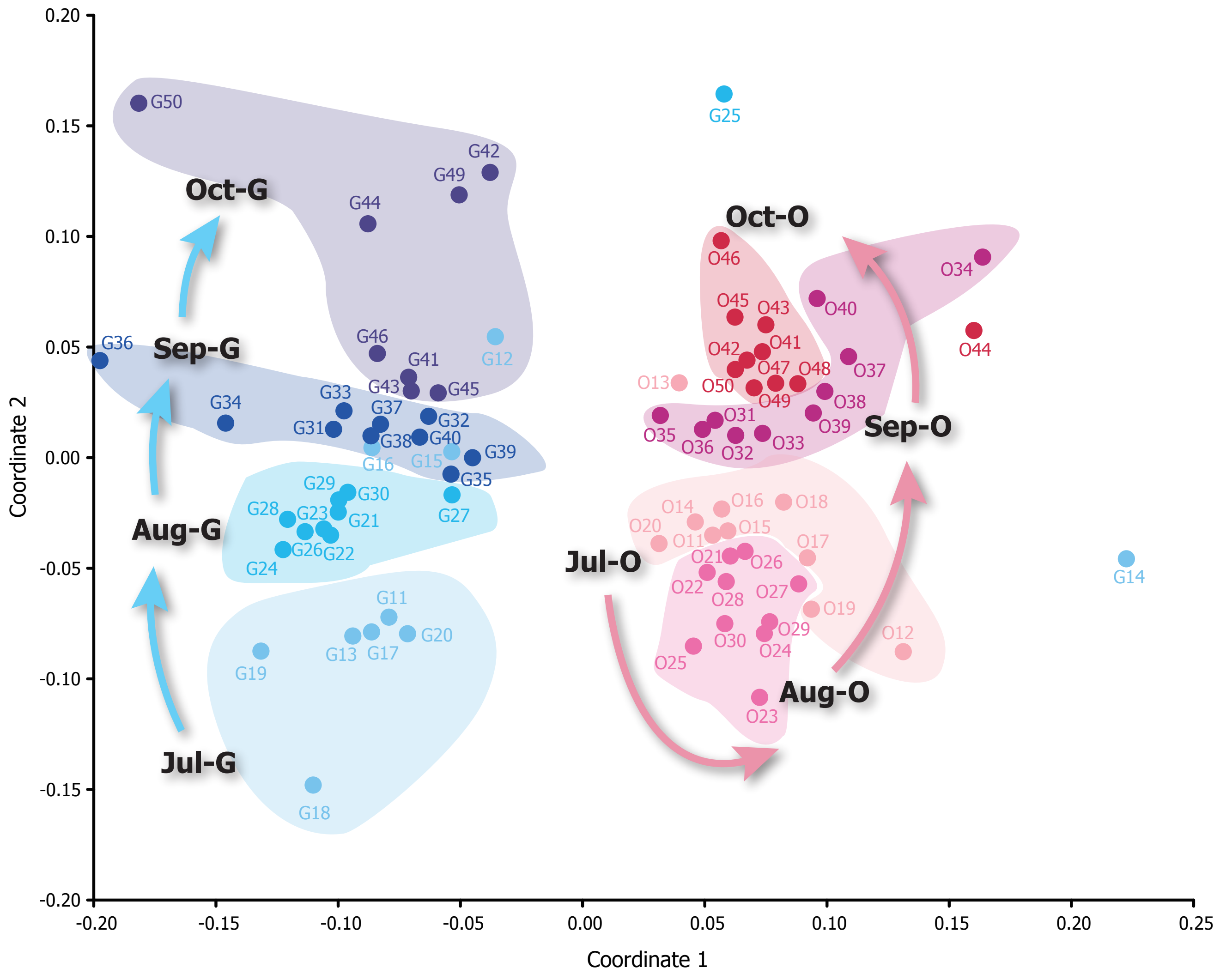

Supplementary Figure S6

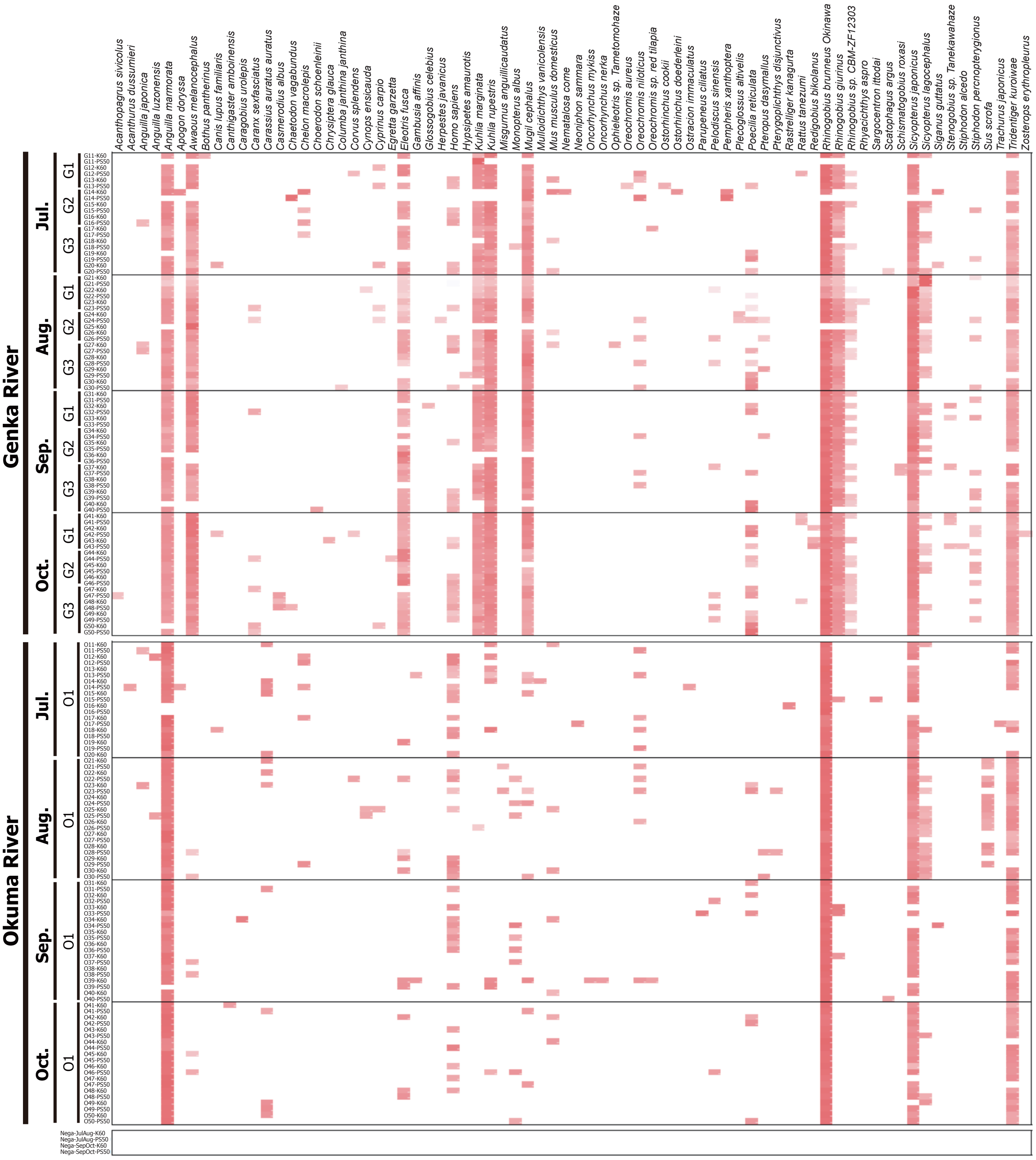

Supplementary Figure S7

	*	*	*	*	*	*	*	*	*	*			*					*
	otu_3017	otu_314	otu_1059	otu_1286	otu_2514	otu_2802	otu_2895	otu_2898	otu_2912	otu_3275	otu_3524	otu_3716	otu_3253	otu_2885	otu_3246	otu_3802	otu_2466	otu_2871
Acanthopagrus sivicolus	-0.16460	-0.09759	-0.09759	-0.09759	-0.09759	-0.09759	-0.09759	-0.09759	-0.09759	-0.04755	-0.12458	-0.12765	-0.11636	-0.19436	-0.17185	-0.15237	-0.25136	0.07972
Acanthurus dussumieri	-0.09715	-0.09759	-0.09759	-0.09759	-0.09759	-0.09759	-0.09759	-0.09759	-0.09759	-0.11496	0.01071	0.11622	-0.08228	-0.19436	0.08573	0.20993	0.01364	-0.18011
Anguilla japonica	0.12684	0.02310	0.02310	0.02310	0.02310	0.02310	0.02310	0.02310	0.02310	-0.00242	-0.00123	-0.00442	-0.06104	0.47459	-0.06423	-0.02639	0.11040	-0.18658
Anguilla luzonensis	0.45592	0.46803	0.46803	0.46803	0.46803	0.46803	0.46803	0.46803	0.46803	0.45342	0.53434	0.58675	0.46870	0.20263	0.55992	0.61618	0.43433	0.10651
Anguilla marmorata	0.45157	0.51672	0.51672	0.51672	0.51672	0.51672	0.51672	0.51672	0.51672	0.49567	0.52838	0.51982	0.41063	0.70503	0.55336	0.54860	0.25063	-0.14913
Apogon doryssa	-0.09737	-0.10629	-0.10629	-0.10629	-0.10629	-0.10629	-0.10629	-0.10629	-0.10629	-0.12521	-0.10040	-0.11778	-0.02451	-0.21169	-0.17158	-0.05748	-0.25068	-0.19617
Awaous melanocephalus	-0.26280	-0.24496	-0.24496	-0.24496	-0.24496	-0.24496	-0.24496	-0.24496	-0.24496	-0.19164	-0.30835	-0.32059	-0.29144	-0.14938	-0.42221	-0.37619	-0.34300	0.11053
Bothus pantherinus	-0.08872	-0.09759	-0.09759	-0.09759	-0.09759	-0.09759	-0.09759	-0.09759	-0.09759	-0.11496	-0.10113	-0.12765	-0.01731	-0.19436	-0.17869	-0.07562	-0.25136	-0.18011
Canis lupus familiaris	-0.21080	-0.17211	-0.17211	-0.17211	-0.17211	-0.17211	-0.17211	-0.17211	-0.17211	-0.15702	-0.15633	-0.14241	-0.11975	-0.34278	-0.22082	-0.08858	-0.35343	-0.14140
Canthigaster amboinensis	-0.16460	-0.09759	-0.09759	-0.09759	-0.09759	-0.09759	-0.09759	-0.09759	-0.09759	-0.11496	-0.10744	-0.12765	-0.15044	0.18978	-0.06733	-0.08013	-0.25136	-0.18011
Caragobius urolepis	-0.12744	-0.09759	-0.09759	-0.09759	-0.09759	-0.09759	-0.09759	-0.09759	-0.09759	-0.05718	-0.10248	-0.12765	-0.08334	-0.19436	-0.00059	-0.17156	-0.03312	-0.18011
Caranx sexfasciatus	-0.14237	-0.18326	-0.18326	-0.18326	-0.18326	-0.18326	-0.18326	-0.18326	-0.18326	-0.16186	-0.23394	-0.23970	-0.25386	0.10219	-0.31252	-0.29254	-0.14104	0.02965
Carassius auratus auratus	0.08686	0.13582	0.13582	0.13582	0.13582	0.13582	0.13582	0.13582	0.13582	0.10735	0.25443	0.35447	0.11888	0.05921	0.36235	0.46914	0.12002	-0.20131
Casmerodius albus	-0.19389	-0.11496	-0.11496	-0.11496	-0.11496	-0.11496	-0.11496	-0.11496	-0.11496	-0.05601	-0.14675	-0.15036	-0.13706	-0.22894	-0.20243	-0.17948	-0.29609	0.09391
Chaetodon vagabundus	-0.10017	-0.10448	-0.10448	-0.10448	-0.10448	-0.10448	-0.10448	-0.10448	-0.10448	-0.11846	-0.10987	-0.13665	-0.02530	-0.20807	-0.19083	-0.08620	-0.26910	-0.17506
Chelon macrolepis	-0.06571	-0.07439	-0.07439	-0.07439	-0.07439	-0.07439	-0.07439	-0.07439	-0.07439	-0.09648	-0.06151	-0.07539	0.01527	-0.21417	-0.13684	-0.00634	-0.23509	-0.20583
Choerodon schoenleinii	-0.01690	-0.09759	-0.09759	-0.09759	-0.09759	-0.09759	-0.09759	-0.09759	-0.09759	-0.11496	-0.12458	-0.12765	-0.04393	-0.19436	-0.14100	-0.18510	0.21629	0.52852
Chrysiptera glauca	-0.16460	-0.09759	-0.09759	-0.09759	-0.09759	-0.09759	-0.09759	-0.09759	-0.09759	-0.04755	-0.12458	-0.12765	-0.11636	-0.19436	-0.17185	-0.15237	-0.25136	0.07972
Columba janthina janthina	-0.00785	-0.09759	-0.09759	-0.09759	-0.09759	-0.09759	-0.09759	-0.09759	-0.09759	-0.11496	-0.12458	-0.12765	-0.17707	0.35442	-0.16805	-0.15124	0.01364	-0.18011
Corvus splendens	0.43393	0.45539	0.45539	0.45539	0.45539	0.45539	0.45539	0.45539	0.45539	0.45207	0.43994	0.40241	0.49444	0.16770	0.35272	0.39679	0.20947	0.13024
Cynops ensicauda	0.76688	0.73767	0.73767	0.73767	0.73767	0.73767	0.73767	0.73767	0.73767	0.72429	0.71292	0.68309	0.68124	0.68178	0.65257	0.62024	0.63540	0.24238
Cyprinus carpio	0.05826	0.01005	0.01005	0.01005	0.01005	0.01005	0.01005	0.01005	0.01005	-0.00601	-0.02418	-0.06218	0.02580	0.12736	-0.15994	-0.04671	-0.15869	-0.20034
Egretta garzetta	-0.16460	-0.09759	-0.09759	-0.09759	-0.09759	-0.09759	-0.09759	-0.09759	-0.09759	-0.04755	-0.12458	-0.12765	-0.11636	-0.19436	-0.17185	-0.15237	-0.25136	0.07972
Eleotris fusca	-0.28407	-0.25343	-0.25343	-0.25343	-0.25343	-0.25343	-0.25343	-0.25343	-0.25343	-0.21217	-0.30824	-0.33470	-0.23116	-0.32455	-0.44619	-0.35144	-0.45005	0.05807
Gambusia affinis	-0.15820	-0.14064	-0.14064	-0.14064	-0.14064	-0.14064	-0.14064	-0.14064	-0.14064	-0.13097	-0.05253	0.02105	-0.11921	-0.28010	0.07172	0.07347	-0.00842	-0.25957
Glossogobius celebius	-0.01690	-0.09759	-0.09759	-0.09759	-0.09759	-0.09759	-0.09759	-0.09759	-0.09759	-0.11496	-0.12458	-0.12765	-0.04393	-0.19436	-0.14100	-0.18510	0.21629	0.52852
Herpestes javanicus	-0.00785	-0.09759	-0.09759	-0.09759	-0.09759	-0.09759	-0.09759	-0.09759	-0.09759	-0.11496	-0.12458	-0.12765	-0.17707	0.35442	-0.16805	-0.15124	0.01364	-0.18011
Homo sapiens	0.13850	0.11123	0.11123	0.11123	0.11123	0.11123	0.11123	0.11123	0.11123	0.10667	0.15161	0.20016	0.04927	0.25843	0.15183	0.22356	0.11493	-0.23635
Hypsipetes amaurotis	-0.00785	-0.09759	-0.09759	-0.09759	-0.09759	-0.09759	-0.09759	-0.09759	-0.09759	-0.11496	-0.12458	-0.12765	-0.17707	0.35442	-0.16805	-0.15124	0.01364	-0.18011
Kuhlia marginata	-0.28136	-0.32304	-0.32304	-0.32304	-0.32304	-0.32304	-0.32304	-0.32304	-0.32304	-0.29702	-0.39645	-0.42286	-0.31630	-0.23307	-0.55179	-0.47201	-0.33668	0.17698
Kuhlia rupestris	-0.23227	-0.30467	-0.30467	-0.30467	-0.30467	-0.30467	-0.30467	-0.30467	-0.30467	-0.29123	-0.37286	-0.39274	-0.33892	-0.01838	-0.51436	-0.43988	-0.26985	0.03602
Misgurnus anguillicaudatus	0.66727	0.68313	0.68313	0.68313	0.68313	0.68313	0.68313	0.68313	0.68313	0.67951	0.67408	0.64967	0.67072	0.42759	0.64177	0.60609	0.54364	0.29231
Monopterus albus	0.26182	0.32455	0.32455	0.32455	0.32455	0.32455	0.32455	0.32455	0.32455	0.33696	0.30947	0.26048	0.31562	0.21266	0.36057	0.23630	0.16491	-0.05241
Mugil cephalus	-0.13587	-0.26094	-0.26094	-0.26094	-0.26094	-0.26094	-0.26094	-0.26094	-0.26094	-0.27210	-0.32501	-0.34189	-0.32166	0.20604	-0.44378	-0.39447	-0.10280	-0.01302
Mulloidichthys vanicolensis	-0.09715	-0.09759	-0.09759	-0.09759	-0.09759	-0.09759	-0.09759	-0.09759	-0.09759	-0.11496	0.01071	0.11622	-0.08228	-0.19436	0.08573	0.20993	0.01364	-0.18011
Mus musculus domesticus	-0.06439	-0.07072	-0.07072	-0.07072	-0.07072	-0.07072	-0.07072	-0.07072	-0.07072	-0.08922	-0.07352	-0.10186	0.00726	-0.16481	-0.14887	-0.04834	-0.23788	-0.19559
Nematalosa come	-0.08872	-0.09759	-0.09759	-0.09759	-0.09759	-0.09759	-0.09759	-0.09759	-0.09759	-0.11496	-0.10113	-0.12765	-0.01731	-0.19436	-0.17869	-0.07562	-0.25136	-0.18011
Neoniphon sammara	-0.09715	-0.09759	-0.09759	-0.09759	-0.09759	-0.09759	-0.09759	-0.09759	-0.09759	-0.11496	0.01071	0.11622	-0.08228	-0.19436	0.08573	0.20993	0.01364	-0.18011
Oncorhynchus mykiss	-0.12744	-0.09759	-0.09759	-0.09759	-0.09759	-0.09759	-0.09759	-0.09759	-0.09759	-0.05718	-0.10248	-0.12765	-0.08334	-0.19436	-0.00059	-0.17156	-0.03312	-0.18011
Oncorhynchus nerka	-0.12744	-0.09759	-0.09759	-0.09759	-0.09759	-0.09759	-0.09759	-0.09759	-0.09759	-0.05718	-0.10248	-0.12765	-0.08334	-0.19436	-0.00059	-0.17156	-0.03312	-0.18011
Ophieleotris sp. Tametomohaze	-0.00785	-0.09759	-0.09759	-0.09759	-0.09759	-0.09759	-0.09759	-0.09759	-0.09759	-0.11496	-0.12458	-0.12765	-0.17707	0.35442	-0.16805	-0.15124	0.01364	-0.18011
Oreochromis aureus	-0.08872	-0.09759	-0.09759	-0.09759	-0.09759	-0.09759	-0.09759	-0.09759	-0.09759	-0.11496	-0.10113	-0.12765	-0.01731	-0.19436	-0.17869	-0.07562	-0.25136	-0.18011
Oreochromis niloticus	0.02207	-0.03963	-0.03963	-0.03963	-0.03963	-0.03963	-0.03963	-0.03963	-0.03963	-0.07426	-0.00106	0.02863	-0.00741	0.00576	-0.05018	0.09226	-0.03474	-0.23358
Oreochromis sp. red tilapia	-0.12917	-0.12745	-0.12745	-0.12745	-0.12745	-0.12745	-0.12745	-0.12745	-0.12745	-0.13027	-0.13253	-0.16670	-0.04530	-0.25382	-0.17215	-0.13173	-0.25326	-0.23522
Ostorhinchus cookii	-0.08872	-0.09759	-0.09759	-0.09759	-0.09759	-0.09759	-0.09759	-0.09759	-0.09759	-0.11496	-0.10113	-0.12765	-0.01731	-0.19436	-0.17869	-0.07562	-0.25136	-0.18011
Ostorhinchus doederleini	-0.08872	-0.09759	-0.09759	-0.09759	-0.09759	-0.09759	-0.09759	-0.09759	-0.09759	-0.11496	-0.10113	-0.12765	-0.01731	-0.19436	-0.17869	-0.07562	-0.25136	-0.18011
Ostracion immaculatus	-0.09715	-0.09759	-0.09759	-0.09759	-0.09759	-0.09759	-0.09759	-0.09759	-0.09759	-0.11496	0.01071	0.11622	-0.08228	-0.19436	0.08573	0.20993	0.01364	-0.18011
Parupeneus ciliatus	-0.12744	-0.09759	-0.09759	-0.09759	-0.09759	-0.09759	-0.09759	-0.09759	-0.09759	-0.05718	-0.10248	-0.12765	-0.08334	-0.19436	-0.00059	-0.17156	-0.03312	-0.18011
Pelodiscus sinensis	-0.17102	-0.21921	-0.21921	-0.21921	-0.21921	-0.21921	-0.21921	-0.21921	-0.21921	-0.21245	-0.26986	-0.28672	-0.31112	0.23945	-0.30701	-0.32995	-0.14923	-0.12476
Pempheris xanthoptera	-0.11681	-0.12849	-0.12849	-0.12849	-0.12849	-0.12849	-0.12849	-0.12849	-0.12849	-0.15136	-0.13315	-0.16806	-0.02279	-0.25590	-0.23527	-0.09956	-0.33095	-0.23714
Plecoglossus altivelis	-0.01131	-0.14064	-0.14064	-0.14064	-0.14064	-0.14064	-0.14064	-0.14064	-0.14064	-0.16567	-0.17954	-0.18396	-0.25518	0.51077	-0.24218	-0.21796	0.01966	-0.25957
Poecilia reticulata	-0.21813	-0.19357	-0.19357	-0.19357	-0.19357	-0.19357	-0.19357	-0.19357	-0.19357	-0.14291	-0.24918	-0.25908	-0.23222	-0.15535	-0.33708	-0.31561	-0.26517	0.16640
Pteropus dasymallus	0.58037	0.50437	0.50437	0.50437	0.50437	0.50437	0.50437	0.50437	0.50437	0.48338	0.46872	0.44398	0.43988	0.62633	0.40128	0.37417	0.53737	0.22258
Pterygoplichthys disjunctivus	0.66727	0.68313	0.68313	0.68313	0.68313	0.68313	0.68313	0.68313	0.68313	0.67951	0.67408	0.64967	0.67072	0.42759	0.64177	0.60609	0.54364	0.29231
Rastrelliger kanagurta	-0.09715	-0.09759	-0.09759	-0.09759	-0.09759	-0.09759	-0.09759	-0.09759	-0.09759	-0.11496	0.01071	0.11622	-0.08228	-0.19436	0.08573	0.20993	0.01364	-0.18011
Rattus tanezumi	-0.20897	-0.16389	-0.16389	-0.16389	-0.16389	-0.16389	-0.16389	-0.16389	-0.16389	-0.13979	-0.18837	-0.21437	-0.10735	-0.32641	-0.29469	-0.18765	-0.42214	-0.09713
Redigobius bikolanus	-0.24066	-0.14268	-0.14268	-0.14268	-0.14268	-0.14268	-0.14268	-0.14268	-0.14268	-0.06952	-0.18214	-0.18663	-0.17012	-0.28416	-0.25125	-0.22277	-0.36750	0.11656
Rhinogobius brunneus Okinawa	-0.05260	-0.13186	-0.13186	-0.13186	-0.13186	-0.13186	-0.13186	-0.13186	-0.13186	-0.12725	-0.19809	-0.22675	-0.15440	0.08339	-0.35570	-0.27317	-0.14077	0.05508
Rhinogobius giurinus	-0.21006	-0.30020	-0.30020	-0.30020	-0.30020	-0.30020	-0.30020	-0.30020	-0.30020	-0.28576	-0.37564	-0.39129	-0.34038	-0.02164	-0.49698	-0.47026	-0.13809	0.22990
Rhinogobius sp. CBM-ZF12303	-0.19320	-0.25620	-0.25620	-0.25620	-0.25620	-0.25620	-0.25620	-0.25620	-0.25620	-0.23554	-0.32295	-0.33511	-0.31270	0.02909	-0.43366	-0.40133	-0.17279	0.12597
Rhyacichthys aspro	-0.00785	-0.09759	-0.09759	-0.09759	-0.09759	-0.09759	-0.09759	-0.09759	-0.09759	-0.11496	-0.12458	-0.12765	-0.17707	0.35442	-0.16805	-0.15124	0.01364	-0.18011
Sargocentron ittodai	-0.09715	-0.09759	-0.09759	-0.09759	-0.09759	-0.09759	-0.09759	-0.09759	-0.09759	-0.11496	0.01071	0.11622	-0.08228	-0.19436	0.08573	0.20993	0.01364	-0.18011
Scatophagus argus	-0.16026	-0.13970	-0.13970	-0.13970	-0.13970	-0.13970	-0.13970	-0.13970	-0.13970	-0.11493	-0.14593	-0.18273	-0.08149	-0.27822	-0.10282	-0.19065	-0.17238	-0.25783
Schismatogobius roxasi	-0.02182	-0.12599	-0.12599	-0.12599	-0.12599	-0.12599	-0.12599	-0.12599	-0.12599	-0.14841	-0.16083	-0.16479	-0.05672	-0.25091	-0.18203	-0.23896	0.27922	0.68232
Sicyopterus japonicus	-0.02120	-0.14481	-0.14481	-0.14481	-0.14481	-0.14481	-0.14481	-0.14481	-0.14481	-0.16553	-0.18771	-0.19455	-0.25248	0.46795	-0.26026	-0.23165	-0.00082	-0.22482
Sicyopterus lagocephalus	-0.01043	-0.13988	-0.13988	-0.13988	-0.13988	-0.13988	-0.13988	-0.13988	-0.13988	-0.16494	-0.17890	-0.18342	-0.25431	0.51100	-0.24192	-0.21753	0.02025	-0.25834
Siganus guttatus	-0.10021	-0.10645	-0.10645	-0.10645	-0.10645	-0.10645	-0.10645	-0.10645	-0.10645	-0.12026	-0.11043	-0.13923	-0.02474	-0.21200	-0.17910	-0.09100	-0.25480	-0.19646
Stenogobius sp. Tanekawahaze	-0.16196	-0.16809	-0.16809	-0.16809	-0.16809	-0.16809	-0.16809	-0.16809	-0.16809	-0.13737	-0.21457	-0.21986	-0.14081	-0.33476	-0.27061	-0.28937	-0.04810	0.50663
Stiphodon alcedo	-0.16460	-0.09759	-0.09759	-0.09759	-0.09759	-0.09759	-0.09759	-0.09759	-0.09759	-0.04755	-0.12458	-0.12765	-0.11636	-0.19436	-0.17185	-0.15237	-0.25136	0.07972
Stiphodon percnopterygionus	-0.19644	-0.27034	-0.27034	-0.27034	-0.27034	-0.27034	-0.27034	-0.27034	-0.27034	-0.25400	-0.33994	-0.35361	-0.29441	-0.08773	-0.45062	-0.42950	-0.11756	0.29670
Sus scrofa	0.97008	0.99314	0.99314	0.99314	0.99314	0.99314	0.99314	0.99314	0.99314	0.98787	0.97998	0.94449	0.97510	0.62163	0.93301	0.88113	0.79034	0.42496
Trachurus japonicus	-0.09715	-0.09759	-0.09759	-0.09759	-0.09759	-0.09759	-0.09759	-0.09759	-0.09759	-0.11496	0.01071	0.11622	-0.08228	-0.19436	0.08573	0.20993	0.01364	-0.18011
Tridentiger kuroiwae	0.04363	0.01434	0.01434	0.01434	0.01434	0.01434	0.01434	0.01434	0.01434	0.04977	-0.06630	-0.10542	-0.04013	0.13178	-0.16581	-0.20607	0.01544	0.24010
Zosterops erythropleurus	-0.16460	-0.09759	-0.09759	-0.09759	-0.09759	-0.09759	-0.09759	-0.09759	-0.09759	-0.04755	-0.12458	-0.12765	-0.11636	-0.19436	-0.17185	-0.15237	-0.25136	0.07972

Supplementary Figure S8

	*	*	*	*	*	*	*	*	*	*			*					*
	otu_3017	otu_314	otu_1059	otu_1286	otu_2514	otu_2802	otu_2895	otu_2898	otu_2912	otu_3275	otu_3524	otu_3716	otu_3253	otu_2885	otu_3246	otu_3802	otu_2466	otu_2871
Acanthopagrus sivicolus	-0.44493	-0.27663	-0.27663	-0.27663	-0.27663	-0.27663	-0.27663	-0.27663	-0.27663	-0.16040	-0.27359	-0.23998	-0.30608	-0.23492	-0.30600	-0.22464	-0.48753	0.09933
Acanthurus dussumieri	0.25786	0.25522	0.25522	0.25522	0.25522	0.25522	0.25522	0.25522	0.25522	0.22648	0.40231	0.51275	0.27365	-0.10010	0.44057	0.50730	0.36957	-0.03569
Anguilla japonica	0.37454	0.11527	0.11527	0.11527	0.11527	0.11527	0.11527	0.11527	0.11527	0.05508	0.04134	0.02794	-0.08938	0.56698	-0.07298	-0.01219	0.24343	-0.24284
Anguilla luzonensis	0.37770	0.40875	0.40875	0.40875	0.40875	0.40875	0.40875	0.40875	0.40875	0.38097	0.50451	0.56334	0.39781	0.03476	0.50698	0.55850	0.28801	-0.21554
Anguilla marmorata	0.40654	0.57643	0.57643	0.57643	0.57643	0.57643	0.57643	0.57643	0.57643	0.54005	0.52084	0.46439	0.29063	0.66308	0.51700	0.47404	-0.04333	-0.58467
Apogon doryssa	0.25301	0.22883	0.22883	0.22883	0.22883	0.22883	0.22883	0.22883	0.22883	0.19460	0.17290	0.09916	0.40880	-0.12177	-0.00027	0.12759	-0.13300	-0.06132
Awaous melanocephalus	-0.54546	-0.49886	-0.49886	-0.49886	-0.49886	-0.49886	-0.49886	-0.49886	-0.49886	-0.38378	-0.53045	-0.48301	-0.58361	-0.14518	-0.63884	-0.47367	-0.55887	0.21006
Bothus pantherinus	0.22914	0.20534	0.20534	0.20534	0.20534	0.20534	0.20534	0.20534	0.20534	0.17380	0.13702	0.05417	0.38261	-0.11236	-0.03841	0.08289	-0.16418	-0.05785
Canis lupus familiaris	-0.04980	0.04822	0.04822	0.04822	0.04822	0.04822	0.04822	0.04822	0.04822	0.09550	0.04900	0.04771	0.16669	-0.28090	-0.09179	0.07800	-0.33584	0.01352
Canthigaster amboinensis	-0.06125	0.10705	0.10705	0.10705	0.10705	0.10705	0.10705	0.10705	0.10705	0.06996	0.05016	-0.00556	-0.02803	0.31513	0.09273	0.03548	-0.22901	-0.10173
Caragobius urolepis	-0.11248	-0.03776	-0.03776	-0.03776	-0.03776	-0.03776	-0.03776	-0.03776	-0.03776	0.06808	-0.04918	-0.09380	-0.00427	-0.17325	0.11923	-0.15170	0.07894	-0.16689
Caranx sexfasciatus	-0.23904	-0.33974	-0.33974	-0.33974	-0.33974	-0.33974	-0.33974	-0.33974	-0.33974	-0.29989	-0.37803	-0.34061	-0.48967	0.14915	-0.45281	-0.35573	-0.18165	0.09586
Carassius auratus auratus	0.36426	0.48436	0.48436	0.48436	0.48436	0.48436	0.48436	0.48436	0.48436	0.43575	0.61746	0.69895	0.41772	0.10719	0.68973	0.70996	0.32209	-0.22404
Casmerodius albus	-0.52413	-0.32587	-0.32587	-0.32587	-0.32587	-0.32587	-0.32587	-0.32587	-0.32587	-0.18895	-0.32229	-0.28270	-0.36056	-0.27674	-0.36047	-0.26462	-0.57431	0.11701
Chaetodon vagabundus	0.19863	0.18650	0.18650	0.18650	0.18650	0.18650	0.18650	0.18650	0.18650	0.16300	0.11825	0.03756	0.36210	-0.12896	-0.05981	0.06742	-0.19849	-0.05105
Chelon macrolepis	0.31023	0.28650	0.28650	0.28650	0.28650	0.28650	0.28650	0.28650	0.28650	0.24657	0.23428	0.15926	0.48224	-0.13040	0.04545	0.18988	-0.11885	-0.08585
Choerodon schoenleinii	-0.31921	-0.51936	-0.51936	-0.51936	-0.51936	-0.51936	-0.51936	-0.51936	-0.51936	-0.59375	-0.45755	-0.38933	-0.36233	-0.29942	-0.39737	-0.37248	0.21413	0.64225
Chrysiptera glauca	-0.44493	-0.27663	-0.27663	-0.27663	-0.27663	-0.27663	-0.27663	-0.27663	-0.27663	-0.16040	-0.27359	-0.23998	-0.30608	-0.23492	-0.30600	-0.22464	-0.48753	0.09933
Columba janthina janthina	0.22342	-0.00177	-0.00177	-0.00177	-0.00177	-0.00177	-0.00177	-0.00177	-0.00177	-0.04510	-0.06616	-0.07184	-0.19228	0.47741	-0.13961	-0.10866	0.19050	-0.15064
Corvus splendens	0.25532	0.31232	0.31232	0.31232	0.31232	0.31232	0.31232	0.31232	0.31232	0.31015	0.25741	0.18821	0.40441	-0.02611	0.10237	0.21084	-0.20147	-0.21193
Cynops ensicauda	0.52304	0.42526	0.42526	0.42526	0.42526	0.42526	0.42526	0.42526	0.42526	0.38062	0.36524	0.32595	0.24617	0.53321	0.26778	0.29808	0.18627	-0.46099
Cyprinus carpio	0.32792	0.20630	0.20630	0.20630	0.20630	0.20630	0.20630	0.20630	0.20630	0.17641	0.08883	0.00418	0.23088	0.19582	-0.16083	0.01119	-0.17006	-0.20702
Egretta garzetta	-0.44493	-0.27663	-0.27663	-0.27663	-0.27663	-0.27663	-0.27663	-0.27663	-0.27663	-0.16040	-0.27359	-0.23998	-0.30608	-0.23492	-0.30600	-0.22464	-0.48753	0.09933
Eleotris fusca	-0.43135	-0.35309	-0.35309	-0.35309	-0.35309	-0.35309	-0.35309	-0.35309	-0.35309	-0.25981	-0.40500	-0.40616	-0.28486	-0.30689	-0.58357	-0.37110	-0.64535	0.21307
Gambusia affinis	0.14857	0.19233	0.19233	0.19233	0.19233	0.19233	0.19233	0.19233	0.19233	0.23319	0.30953	0.37535	0.22851	-0.19116	0.44549	0.33512	0.36072	-0.13285
Glossogobius celebius	-0.31921	-0.51936	-0.51936	-0.51936	-0.51936	-0.51936	-0.51936	-0.51936	-0.51936	-0.59375	-0.45755	-0.38933	-0.36233	-0.29942	-0.39737	-0.37248	0.21413	0.64225
Herpestes javanicus	0.22342	-0.00177	-0.00177	-0.00177	-0.00177	-0.00177	-0.00177	-0.00177	-0.00177	-0.04510	-0.06616	-0.07184	-0.19228	0.47741	-0.13961	-0.10866	0.19050	-0.15064
Homo sapiens	0.35645	0.28719	0.28719	0.28719	0.28719	0.28719	0.28719	0.28719	0.28719	0.28989	0.30970	0.35004	0.12636	0.30331	0.25905	0.31311	0.21975	-0.33604
Hypsipetes amaurotis	0.22342	-0.00177	-0.00177	-0.00177	-0.00177	-0.00177	-0.00177	-0.00177	-0.00177	-0.04510	-0.06616	-0.07184	-0.19228	0.47741	-0.13961	-0.10866	0.19050	-0.15064
Kuhlia marginata	-0.43525	-0.53780	-0.53780	-0.53780	-0.53780	-0.53780	-0.53780	-0.53780	-0.53780	-0.49402	-0.58955	-0.56485	-0.49668	-0.20248	-0.76652	-0.54298	-0.44168	0.38077
Kuhlia rupestris	-0.23316	-0.41431	-0.41431	-0.41431	-0.41431	-0.41431	-0.41431	-0.41431	-0.41431	-0.39640	-0.48452	-0.46629	-0.47800	0.06994	-0.66042	-0.46729	-0.26438	0.21335
Misgurnus anguillicaudatus	0.30197	0.35263	0.35263	0.35263	0.35263	0.35263	0.35263	0.35263	0.35263	0.34103	0.34061	0.31144	0.31582	0.16205	0.30292	0.30991	0.04276	-0.29317
Monopterus albus	0.10186	0.26239	0.26239	0.26239	0.26239	0.26239	0.26239	0.26239	0.26239	0.30359	0.20458	0.11215	0.23302	0.10663	0.28590	0.09839	-0.07415	-0.33815
Mugil cephalus	-0.00633	-0.32038	-0.32038	-0.32038	-0.32038	-0.32038	-0.32038	-0.32038	-0.32038	-0.36278	-0.40016	-0.38761	-0.45165	0.32931	-0.55054	-0.41026	0.03651	0.13413
Mulloidichthys vanicolensis	0.25786	0.25522	0.25522	0.25522	0.25522	0.25522	0.25522	0.25522	0.25522	0.22648	0.40231	0.51275	0.27365	-0.10010	0.44057	0.50730	0.36957	-0.03569
Mus musculus domesticus	0.25982	0.24238	0.24238	0.24238	0.24238	0.24238	0.24238	0.24238	0.24238	0.20952	0.16960	0.08044	0.41206	-0.08504	-0.00557	0.10829	-0.15913	-0.09429
Nematalosa come	0.22914	0.20534	0.20534	0.20534	0.20534	0.20534	0.20534	0.20534	0.20534	0.17380	0.13702	0.05417	0.38261	-0.11236	-0.03841	0.08289	-0.16418	-0.05785
Neoniphon sammara	0.25786	0.25522	0.25522	0.25522	0.25522	0.25522	0.25522	0.25522	0.25522	0.22648	0.40231	0.51275	0.27365	-0.10010	0.44057	0.50730	0.36957	-0.03569
Oncorhynchus mykiss	-0.11248	-0.03776	-0.03776	-0.03776	-0.03776	-0.03776	-0.03776	-0.03776	-0.03776	0.06808	-0.04918	-0.09380	-0.00427	-0.17325	0.11923	-0.15170	0.07894	-0.16689
Oncorhynchus nerka	-0.11248	-0.03776	-0.03776	-0.03776	-0.03776	-0.03776	-0.03776	-0.03776	-0.03776	0.06808	-0.04918	-0.09380	-0.00427	-0.17325	0.11923	-0.15170	0.07894	-0.16689
Ophieleotris sp. Tametomohaze	0.22342	-0.00177	-0.00177	-0.00177	-0.00177	-0.00177	-0.00177	-0.00177	-0.00177	-0.04510	-0.06616	-0.07184	-0.19228	0.47741	-0.13961	-0.10866	0.19050	-0.15064
Oreochromis aureus	0.22914	0.20534	0.20534	0.20534	0.20534	0.20534	0.20534	0.20534	0.20534	0.17380	0.13702	0.05417	0.38261	-0.11236	-0.03841	0.08289	-0.16418	-0.05785
Oreochromis niloticus	0.47924	0.32107	0.32107	0.32107	0.32107	0.32107	0.32107	0.32107	0.32107	0.24943	0.31621	0.30814	0.37715	0.11667	0.16150	0.30568	0.22240	-0.14997
Oreochromis sp. red tilapia	0.18217	0.18536	0.18536	0.18536	0.18536	0.18536	0.18536	0.18536	0.18536	0.19199	0.11527	0.01949	0.36857	-0.16945	0.00467	0.02696	-0.13111	-0.11450
Ostorhinchus cookii	0.22914	0.20534	0.20534	0.20534	0.20534	0.20534	0.20534	0.20534	0.20534	0.17380	0.13702	0.05417	0.38261	-0.11236	-0.03841	0.08289	-0.16418	-0.05785
Ostorhinchus doederleini	0.22914	0.20534	0.20534	0.20534	0.20534	0.20534	0.20534	0.20534	0.20534	0.17380	0.13702	0.05417	0.38261	-0.11236	-0.03841	0.08289	-0.16418	-0.05785
Ostracion immaculatus	0.25786	0.25522	0.25522	0.25522	0.25522	0.25522	0.25522	0.25522	0.25522	0.22648	0.40231	0.51275	0.27365	-0.10010	0.44057	0.50730	0.36957	-0.03569
Parupeneus ciliatus	-0.11248	-0.03776	-0.03776	-0.03776	-0.03776	-0.03776	-0.03776	-0.03776	-0.03776	0.06808	-0.04918	-0.09380	-0.00427	-0.17325	0.11923	-0.15170	0.07894	-0.16689
Pelodiscus sinensis	-0.10595	-0.22659	-0.22659	-0.22659	-0.22659	-0.22659	-0.22659	-0.22659	-0.22659	-0.21714	-0.29753	-0.29856	-0.43667	0.36542	-0.32683	-0.32467	-0.05763	-0.03391
Pempheris xanthoptera	0.30618	0.27437	0.27437	0.27437	0.27437	0.27437	0.27437	0.27437	0.27437	0.23224	0.18308	0.07238	0.51125	-0.15014	-0.05132	0.11076	-0.21938	-0.07730
Plecoglossus altivelis	0.32397	-0.00257	-0.00257	-0.00257	-0.00257	-0.00257	-0.00257	-0.00257	-0.00257	-0.06540	-0.09594	-0.10417	-0.27881	0.69226	-0.20244	-0.15756	0.27623	-0.21843
Poecilia reticulata	-0.55152	-0.48818	-0.48818	-0.48818	-0.48818	-0.48818	-0.48818	-0.48818	-0.48818	-0.38076	-0.50103	-0.44898	-0.55369	-0.18260	-0.56521	-0.43868	-0.49474	0.23664
Pteropus dasymallus	0.37116	0.15393	0.15393	0.15393	0.15393	0.15393	0.15393	0.15393	0.15393	0.08662	0.09923	0.08712	-0.01507	0.50132	0.01536	0.05565	0.26238	-0.21925
Pterygoplichthys disjunctivus	0.30197	0.35263	0.35263	0.35263	0.35263	0.35263	0.35263	0.35263	0.35263	0.34103	0.34061	0.31144	0.31582	0.16205	0.30292	0.30991	0.04276	-0.29317
Rastrelliger kanagurta	0.25786	0.25522	0.25522	0.25522	0.25522	0.25522	0.25522	0.25522	0.25522	0.22648	0.40231	0.51275	0.27365	-0.10010	0.44057	0.50730	0.36957	-0.03569
Rattus tanezumi	-0.15381	-0.04008	-0.04008	-0.04008	-0.04008	-0.04008	-0.04008	-0.04008	-0.04008	0.02513	-0.09801	-0.14431	0.09299	-0.28740	-0.27897	-0.10663	-0.53547	0.02842
Redigobius bikolanus	-0.65060	-0.40450	-0.40450	-0.40450	-0.40450	-0.40450	-0.40450	-0.40450	-0.40450	-0.23454	-0.40005	-0.35091	-0.44756	-0.34351	-0.44744	-0.32847	-0.71289	0.14525
Rhinogobius brunneus Okinawa	-0.06195	-0.25857	-0.25857	-0.25857	-0.25857	-0.25857	-0.25857	-0.25857	-0.25857	-0.25892	-0.34179	-0.34677	-0.29892	0.11496	-0.55164	-0.34826	-0.21293	0.11110
Rhinogobius giurinus	-0.38335	-0.60600	-0.60600	-0.60600	-0.60600	-0.60600	-0.60600	-0.60600	-0.60600	-0.59749	-0.64169	-0.58607	-0.67074	0.01148	-0.74601	-0.59127	-0.15938	0.39685
Rhinogobius sp. CBM-ZF12303	-0.33384	-0.48925	-0.48925	-0.48925	-0.48925	-0.48925	-0.48925	-0.48925	-0.48925	-0.45851	-0.53126	-0.48508	-0.59862	0.07251	-0.63627	-0.49297	-0.21854	0.25001
Rhyacichthys aspro	0.22342	-0.00177	-0.00177	-0.00177	-0.00177	-0.00177	-0.00177	-0.00177	-0.00177	-0.04510	-0.06616	-0.07184	-0.19228	0.47741	-0.13961	-0.10866	0.19050	-0.15064
Sargocentron ittodai	0.25786	0.25522	0.25522	0.25522	0.25522	0.25522	0.25522	0.25522	0.25522	0.22648	0.40231	0.51275	0.27365	-0.10010	0.44057	0.50730	0.36957	-0.03569
Scatophagus argus	0.03306	0.08454	0.08454	0.08454	0.08454	0.08454	0.08454	0.08454	0.08454	0.15871	0.03552	-0.05073	0.21510	-0.21518	0.08190	-0.08474	-0.02513	-0.17847
Schismatogobius roxasi	-0.41413	-0.67380	-0.67380	-0.67380	-0.67380	-0.67380	-0.67380	-0.67380	-0.67380	-0.77031	-0.59361	-0.50510	-0.47008	-0.38845	-0.51553	-0.48325	0.27780	0.83323
Sicyopterus japonicus	0.26965	-0.04167	-0.04167	-0.04167	-0.04167	-0.04167	-0.04167	-0.04167	-0.04167	-0.09528	-0.13383	-0.13989	-0.29876	0.63323	-0.24921	-0.18833	0.21797	-0.18050
Sicyopterus lagocephalus	0.32375	-0.00299	-0.00299	-0.00299	-0.00299	-0.00299	-0.00299	-0.00299	-0.00299	-0.05642	-0.09642	-0.10465	-0.27889	0.69181	-0.20335	-0.15792	0.27570	-0.21767
Siganus guttatus	0.21984	0.20269	0.20269	0.20269	0.20269	0.20269	0.20269	0.20269	0.20269	0.18060	0.13309	0.04590	0.38366	-0.12843	-0.02778	0.06950	-0.15766	-0.07314
Stenogobius sp. Tanekawahaze	-0.66516	-0.67739	-0.67739	-0.67739	-0.67739	-0.67739	-0.67739	-0.67739	-0.67739	-0.63335	-0.62381	-0.53731	-0.57494	-0.45880	-0.60367	-0.50957	-0.26523	0.61795
Stiphodon alcedo	-0.44493	-0.27663	-0.27663	-0.27663	-0.27663	-0.27663	-0.27663	-0.27663	-0.27663	-0.16040	-0.27359	-0.23998	-0.30608	-0.23492	-0.30600	-0.22464	-0.48753	0.09933
Stiphodon percnopterygionus	-0.43813	-0.61977	-0.61977	-0.61977	-0.61977	-0.61977	-0.61977	-0.61977	-0.61977	-0.60790	-0.63685	-0.57497	-0.64477	-0.08858	-0.71966	-0.57124	-0.18192	0.45190
Sus scrofa	0.82194	0.95985	0.95985	0.95985	0.95985	0.95985	0.95985	0.95985	0.95985	0.92827	0.92713	0.84773	0.85963	0.44108	0.82454	0.84355	0.11639	-0.79800
Trachurus japonicus	0.25786	0.25522	0.25522	0.25522	0.25522	0.25522	0.25522	0.25522	0.25522	0.22648	0.40231	0.51275	0.27365	-0.10010	0.44057	0.50730	0.36957	-0.03569
Tridentiger kuroiwae	-0.35735	-0.42966	-0.42966	-0.42966	-0.42966	-0.42966	-0.42966	-0.42966	-0.42966	-0.36141	-0.48677	-0.47027	-0.53335	0.03481	-0.55421	-0.48524	-0.28917	0.14029
Zosterops erythropleurus	-0.44493	-0.27663	-0.27663	-0.27663	-0.27663	-0.27663	-0.27663	-0.27663	-0.27663	-0.16040	-0.27359	-0.23998	-0.30608	-0.23492	-0.30600	-0.22464	-0.48753	0.09933

Supplementary Figure S9

	*	*	*	*	*	*	*	*	*	*			*					*
	otu_3017	otu_314	otu_1059	otu_1286	otu_2514	otu_2802	otu_2895	otu_2898	otu_2912	otu_3275	otu_3524	otu_3716	otu_3253	otu_2885	otu_3246	otu_3802	otu_2466	otu_2871
Acanthopagrus sivicolus	0.0732	0.2993	0.2993	0.2993	0.2993	0.2993	0.2993	0.2993	0.2993	0.5579	0.3051	0.3728	0.2464	0.3835	0.2465	0.4059	0.0441	0.7189
Acanthurus dussumieri	0.3359	0.3412	0.3412	0.3412	0.3412	0.3412	0.3412	0.3412	0.3412	0.4018	0.1131	0.0313	0.3050	0.7168	0.0768	0.0338	0.1516	0.8975
Anguilla japonica	0.1453	0.6756	0.6756	0.6756	0.6756	0.6756	0.6756	0.6756	0.6756	0.8424	0.8814	0.9197	0.7463	0.0131	0.7919	0.9649	0.3655	0.3668
Anguilla luzonensis	0.1414	0.1063	0.1063	0.1063	0.1063	0.1063	0.1063	0.1063	0.1063	0.1374	0.0351	0.0140	0.1180	0.9002	0.0339	0.0152	0.2782	0.4261
Anguilla marmorata	0.1086	0.0110	0.0110	0.0110	0.0110	0.0110	0.0110	0.0110	0.0110	0.0207	0.0278	0.0587	0.2735	0.0014	0.0294	0.0522	0.8757	0.0094
Apogon doryssa	0.3457	0.3967	0.3967	0.3967	0.3967	0.3967	0.3967	0.3967	0.3967	0.4744	0.5268	0.7194	0.1063	0.6582	0.9992	0.6428	0.6285	0.8247
Awaous melanocephalus	0.0190	0.0380	0.0380	0.0380	0.0380	0.0380	0.0380	0.0380	0.0380	0.1340	0.0241	0.0467	0.0096	0.5968	0.0028	0.0525	0.0151	0.4385
Bothus pantherinus	0.3960	0.4494	0.4494	0.4494	0.4494	0.4494	0.4494	0.4494	0.4494	0.5246	0.6180	0.8449	0.1354	0.6835	0.8898	0.7643	0.5484	0.8345
Canis lupus familiaris	0.8573	0.8618	0.8618	0.8618	0.8618	0.8618	0.8618	0.8618	0.8618	0.7294	0.8596	0.8633	0.5422	0.2913	0.7396	0.7779	0.1986	0.9611
Canthigaster amboinensis	0.8249	0.6979	0.6979	0.6979	0.6979	0.6979	0.6979	0.6979	0.6979	0.8004	0.8563	0.9840	0.9195	0.2312	0.7370	0.8981	0.3963	0.7124
Caragobius urolepis	0.6832	0.8916	0.8916	0.8916	0.8916	0.8916	0.8916	0.8916	0.8916	0.8056	0.8591	0.7341	0.9877	0.5259	0.6650	0.5800	0.7753	0.5417
Caranx sexfasciatus	0.3748	0.1928	0.1928	0.1928	0.1928	0.1928	0.1928	0.1928	0.1928	0.2570	0.1409	0.1915	0.0429	0.5866	0.0671	0.1699	0.5054	0.7284
Carassius auratus auratus	0.1585	0.0459	0.0459	0.0459	0.0459	0.0459	0.0459	0.0459	0.0459	0.0809	0.0047	0.0004	0.0974	0.6975	0.0006	0.0003	0.2199	0.4072
Casmerodius albus	0.0265	0.2139	0.2139	0.2139	0.2139	0.2139	0.2139	0.2139	0.2139	0.4878	0.2196	0.2879	0.1634	0.2991	0.1635	0.3225	0.0114	0.6710
Chaetodon vagabundus	0.4649	0.4937	0.4937	0.4937	0.4937	0.4937	0.4937	0.4937	0.4937	0.5514	0.6677	0.8922	0.1613	0.6391	0.8290	0.8075	0.4653	0.8538
Chelon macrolepis	0.2394	0.2810	0.2810	0.2810	0.2810	0.2810	0.2810	0.2810	0.2810	0.3590	0.3849	0.5608	0.0472	0.6354	0.8697	0.4856	0.6660	0.7560
Choerodon schoenleinii	0.2246	0.0284	0.0284	0.0284	0.0284	0.0284	0.0284	0.0284	0.0284	0.0078	0.0636	0.1275	0.1610	0.2579	0.1185	0.1479	0.4293	0.0025
Chrysiptera glauca	0.0732	0.2993	0.2993	0.2993	0.2993	0.2993	0.2993	0.2993	0.2993	0.5579	0.3051	0.3728	0.2464	0.3835	0.2465	0.4059	0.0441	0.7189
Columba janthina janthina	0.4086	0.9949	0.9949	0.9949	0.9949	0.9949	0.9949	0.9949	0.9949	0.8707	0.8110	0.7951	0.4799	0.0501	0.6112	0.6935	0.4841	0.5827
Corvus splendens	0.3410	0.2359	0.2359	0.2359	0.2359	0.2359	0.2359	0.2359	0.2359	0.2395	0.3368	0.4896	0.1109	0.9250	0.7106	0.4368	0.4583	0.4343
Cynops ensicauda	0.0269	0.0902	0.0902	0.0902	0.0902	0.0902	0.0902	0.0902	0.0902	0.1378	0.1572	0.2138	0.3598	0.0231	0.3163	0.2602	0.4942	0.0611
Cyprinus carpio	0.2107	0.4472	0.4472	0.4472	0.4472	0.4472	0.4472	0.4472	0.4472	0.5182	0.7478	0.9880	0.3922	0.4715	0.5568	0.9678	0.5338	0.4455
Egretta garzetta	0.0732	0.2993	0.2993	0.2993	0.2993	0.2993	0.2993	0.2993	0.2993	0.5579	0.3051	0.3728	0.2464	0.3835	0.2465	0.4059	0.0441	0.7189
Eleotris fusca	0.0847	0.1736	0.1736	0.1736	0.1736	0.1736	0.1736	0.1736	0.1736	0.3320	0.1102	0.1090	0.2840	0.2450	0.0096	0.1496	0.0023	0.4317
Gambusia affinis	0.5880	0.4798	0.4798	0.4798	0.4798	0.4798	0.4798	0.4798	0.4798	0.3873	0.2405	0.1443	0.3974	0.4826	0.0728	0.1997	0.1632	0.6289
Glossogobius celebius	0.2246	0.0284	0.0284	0.0284	0.0284	0.0284	0.0284	0.0284	0.0284	0.0078	0.0636	0.1275	0.1610	0.2579	0.1185	0.1479	0.4293	0.0025
Herpestes javanicus	0.4086	0.9949	0.9949	0.9949	0.9949	0.9949	0.9949	0.9949	0.9949	0.8707	0.8110	0.7951	0.4799	0.0501	0.6112	0.6935	0.4841	0.5827
Homo sapiens	0.1690	0.2797	0.2797	0.2797	0.2797	0.2797	0.2797	0.2797	0.2797	0.2748	0.2402	0.1779	0.6460	0.2511	0.3335	0.2346	0.4167	0.1983
Hypsipetes amaurotis	0.4086	0.9949	0.9949	0.9949	0.9949	0.9949	0.9949	0.9949	0.9949	0.8707	0.8110	0.7951	0.4799	0.0501	0.6112	0.6935	0.4841	0.5827
Kuhlia marginata	0.0813	0.0214	0.0214	0.0214	0.0214	0.0214	0.0214	0.0214	0.0214	0.0405	0.0085	0.0136	0.0391	0.4560	0.0000	0.0197	0.0759	0.1376
Kuhlia rupestris	0.3873	0.1007	0.1007	0.1007	0.1007	0.1007	0.1007	0.1007	0.1007	0.1195	0.0458	0.0574	0.0497	0.8004	0.0015	0.0567	0.3230	0.4311
Misgurnus anguillicaudatus	0.2534	0.1742	0.1742	0.1742	0.1742	0.1742	0.1742	0.1742	0.1742	0.1909	0.1915	0.2373	0.2301	0.5538	0.2518	0.2399	0.8774	0.2689
Monopterus albus	0.7120	0.3269	0.3269	0.3269	0.3269	0.3269	0.3269	0.3269	0.3269	0.2506	0.4511	0.6841	0.3876	0.6990	0.2820	0.7215	0.7886	0.1951
Mugil cephalus	0.9818	0.2227	0.2227	0.2227	0.2227	0.2227	0.2227	0.2227	0.2227	0.1604	0.1154	0.1295	0.0680	0.2086	0.0174	0.1048	0.8952	0.6255
Mulloidichthys vanicolensis	0.3359	0.3412	0.3412	0.3412	0.3412	0.3412	0.3412	0.3412	0.3412	0.4018	0.1131	0.0313	0.3050	0.7168	0.0768	0.0338	0.1516	0.8975
Mus musculus domesticus	0.3320	0.3677	0.3677	0.3677	0.3677	0.3677	0.3677	0.3677	0.3677	0.4398	0.5349	0.7711	0.1030	0.7583	0.9840	0.6945	0.5611	0.7327
Nematalosa come	0.3960	0.4494	0.4494	0.4494	0.4494	0.4494	0.4494	0.4494	0.4494	0.5246	0.6180	0.8449	0.1354	0.6835	0.8898	0.7643	0.5484	0.8345
Neoniphon sammara	0.3359	0.3412	0.3412	0.3412	0.3412	0.3412	0.3412	0.3412	0.3412	0.4018	0.1131	0.0313	0.3050	0.7168	0.0768	0.0338	0.1516	0.8975
Oncorhynchus mykiss	0.6832	0.8916	0.8916	0.8916	0.8916	0.8916	0.8916	0.8916	0.8916	0.8056	0.8591	0.7341	0.9877	0.5259	0.6650	0.5800	0.7753	0.5417
Oncorhynchus nerka	0.6832	0.8916	0.8916	0.8916	0.8916	0.8916	0.8916	0.8916	0.8916	0.8056	0.8591	0.7341	0.9877	0.5259	0.6650	0.5800	0.7753	0.5417
Ophieleotris sp. Tametomohaze	0.4086	0.9949	0.9949	0.9949	0.9949	0.9949	0.9949	0.9949	0.9949	0.8707	0.8110	0.7951	0.4799	0.0501	0.6112	0.6935	0.4841	0.5827
Oreochromis aureus	0.3960	0.4494	0.4494	0.4494	0.4494	0.4494	0.4494	0.4494	0.4494	0.5246	0.6180	0.8449	0.1354	0.6835	0.8898	0.7643	0.5484	0.8345
Oreochromis niloticus	0.0490	0.2216	0.2216	0.2216	0.2216	0.2216	0.2216	0.2216	0.2216	0.3531	0.2294	0.2429	0.1420	0.6719	0.5552	0.2470	0.4108	0.5845
Oreochromis sp. red tilapia	0.5041	0.4964	0.4964	0.4964	0.4964	0.4964	0.4964	0.4964	0.4964	0.4806	0.6756	0.9440	0.1529	0.5353	0.9866	0.9225	0.6335	0.6777
Ostorhinchus cookii	0.3960	0.4494	0.4494	0.4494	0.4494	0.4494	0.4494	0.4494	0.4494	0.5246	0.6180	0.8449	0.1354	0.6835	0.8898	0.7643	0.5484	0.8345
Ostorhinchus doederleini	0.3960	0.4494	0.4494	0.4494	0.4494	0.4494	0.4494	0.4494	0.4494	0.5246	0.6180	0.8449	0.1354	0.6835	0.8898	0.7643	0.5484	0.8345
Ostracion immaculatus	0.3359	0.3412	0.3412	0.3412	0.3412	0.3412	0.3412	0.3412	0.3412	0.4018	0.1131	0.0313	0.3050	0.7168	0.0768	0.0338	0.1516	0.8975
Parupeneus ciliatus	0.6832	0.8916	0.8916	0.8916	0.8916	0.8916	0.8916	0.8916	0.8916	0.8056	0.8591	0.7341	0.9877	0.5259	0.6650	0.5800	0.7753	0.5417
Pelodiscus sinensis	0.7008	0.4016	0.4016	0.4016	0.4016	0.4016	0.4016	0.4016	0.4016	0.4225	0.2612	0.2594	0.0801	0.1569	0.2124	0.2158	0.8351	0.9026
Pempheris xanthoptera	0.2462	0.3036	0.3036	0.3036	0.3036	0.3036	0.3036	0.3036	0.3036	0.3893	0.5019	0.7936	0.0320	0.5840	0.8530	0.6878	0.4175	0.7798
Plecoglossus altivelis	0.2170	0.9926	0.9926	0.9926	0.9926	0.9926	0.9926	0.9926	0.9926	0.8132	0.7282	0.7057	0.2952	0.0005	0.4561	0.5651	0.3001	0.4196
Poecilia reticulata	0.0171	0.0437	0.0437	0.0437	0.0437	0.0437	0.0437	0.0437	0.0437	0.1376	0.0369	0.0700	0.0165	0.5031	0.0135	0.0784	0.0401	0.3799
Pteropus dasymallus	0.1495	0.5743	0.5743	0.5743	0.5743	0.5743	0.5743	0.5743	0.5743	0.7539	0.7192	0.7525	0.9567	0.0367	0.9558	0.8407	0.3269	0.4178
Pterygoplichthys disjunctivus	0.2534	0.1742	0.1742	0.1742	0.1742	0.1742	0.1742	0.1742	0.1742	0.1909	0.1915	0.2373	0.2301	0.5538	0.2518	0.2399	0.8774	0.2689
Rastrelliger kanagurta	0.3359	0.3412	0.3412	0.3412	0.3412	0.3412	0.3412	0.3412	0.3412	0.4018	0.1131	0.0313	0.3050	0.7168	0.0768	0.0338	0.1516	0.8975
Rattus tanezumi	0.5746	0.8850	0.8850	0.8850	0.8850	0.8850	0.8850	0.8850	0.8850	0.9278	0.7225	0.5990	0.7363	0.2793	0.2949	0.6990	0.0223	0.9183
Redigobius bikolanus	0.0020	0.1108	0.1108	0.1108	0.1108	0.1108	0.1108	0.1108	0.1108	0.3843	0.1155	0.1766	0.0711	0.1872	0.0712	0.2099	0.0002	0.5966
Rhinogobius brunneus Okinawa	0.8229	0.3345	0.3345	0.3345	0.3345	0.3345	0.3345	0.3345	0.3345	0.3338	0.1898	0.1825	0.2587	0.6765	0.0171	0.1804	0.4320	0.6869
Rhinogobius giurinus	0.1345	0.0060	0.0060	0.0060	0.0060	0.0060	0.0060	0.0060	0.0060	0.0072	0.0026	0.0091	0.0011	0.9670	0.0001	0.0082	0.5605	0.1190
Rhinogobius sp. CBM-ZF12303	0.2016	0.0431	0.0431	0.0431	0.0431	0.0431	0.0431	0.0431	0.0431	0.0628	0.0238	0.0455	0.0071	0.7932	0.0029	0.0411	0.4194	0.3518
Rhyacichthys aspro	0.4086	0.9949	0.9949	0.9949	0.9949	0.9949	0.9949	0.9949	0.9949	0.8707	0.8110	0.7951	0.4799	0.0501	0.6112	0.6935	0.4841	0.5827
Sargocentron ittodai	0.3359	0.3412	0.3412	0.3412	0.3412	0.3412	0.3412	0.3412	0.3412	0.4018	0.1131	0.0313	0.3050	0.7168	0.0768	0.0338	0.1516	0.8975
Scatophagus argus	0.9051	0.7597	0.7597	0.7597	0.7597	0.7597	0.7597	0.7597	0.7597	0.5622	0.8980	0.8547	0.4271	0.4269	0.7670	0.7591	0.9278	0.5131
Schismatogobius roxasi	0.1009	0.0010	0.0010	0.0010	0.0010	0.0010	0.0010	0.0010	0.0010	0.0000	0.0078	0.0348	0.0548	0.1285	0.0301	0.0466	0.2971	0.0000
Sicyopterus japonicus	0.3127	0.8805	0.8805	0.8805	0.8805	0.8805	0.8805	0.8805	0.8805	0.7300	0.6263	0.6105	0.2590	0.0032	0.3535	0.4893	0.4207	0.5082
Sicyopterus lagocephalus	0.2173	0.9914	0.9914	0.9914	0.9914	0.9914	0.9914	0.9914	0.9914	0.8117	0.7269	0.7044	0.2950	0.0006	0.4540	0.5642	0.3011	0.4213
Siganus guttatus	0.4165	0.4555	0.4555	0.4555	0.4555	0.4555	0.4555	0.4555	0.4555	0.5079	0.6283	0.8684	0.1342	0.6406	0.9202	0.8017	0.5648	0.7915
Stenogobius sp. Tanekawahaze	0.0013	0.0009	0.0009	0.0009	0.0009	0.0009	0.0009	0.0009	0.0009	0.0032	0.0040	0.0216	0.0113	0.0626	0.0063	0.0327	0.3213	0.0046
Stiphodon alcedo	0.0732	0.2993	0.2993	0.2993	0.2993	0.2993	0.2993	0.2993	0.2993	0.5579	0.3051	0.3728	0.2464	0.3835	0.2465	0.4059	0.0441	0.7189
Stiphodon percnopterygionus	0.0789	0.0044	0.0044	0.0044	0.0044	0.0044	0.0044	0.0044	0.0044	0.0058	0.0029	0.0113	0.0024	0.7485	0.0002	0.0121	0.5047	0.0678
Sus scrofa	0.0000	0.0000	0.0000	0.0000	0.0000	0.0000	0.0000	0.0000	0.0000	0.0000	0.0000	0.0000	0.0000	0.0764	0.0000	0.0000	0.6726	0.0000
Trachurus japonicus	0.3359	0.3412	0.3412	0.3412	0.3412	0.3412	0.3412	0.3412	0.3412	0.4018	0.1131	0.0313	0.3050	0.7168	0.0768	0.0338	0.1516	0.8975
Tridentiger kuroiwae	0.1677	0.0862	0.0862	0.0862	0.0862	0.0862	0.0862	0.0862	0.0862	0.1622	0.0445	0.0547	0.0230	0.9001	0.0164	0.0454	0.2761	0.6094
Zosterops erythropleurus	0.0732	0.2993	0.2993	0.2993	0.2993	0.2993	0.2993	0.2993	0.2993	0.5579	0.3051	0.3728	0.2464	0.3835	0.2465	0.4059	0.0441	0.7189

Supplementary Figure S10

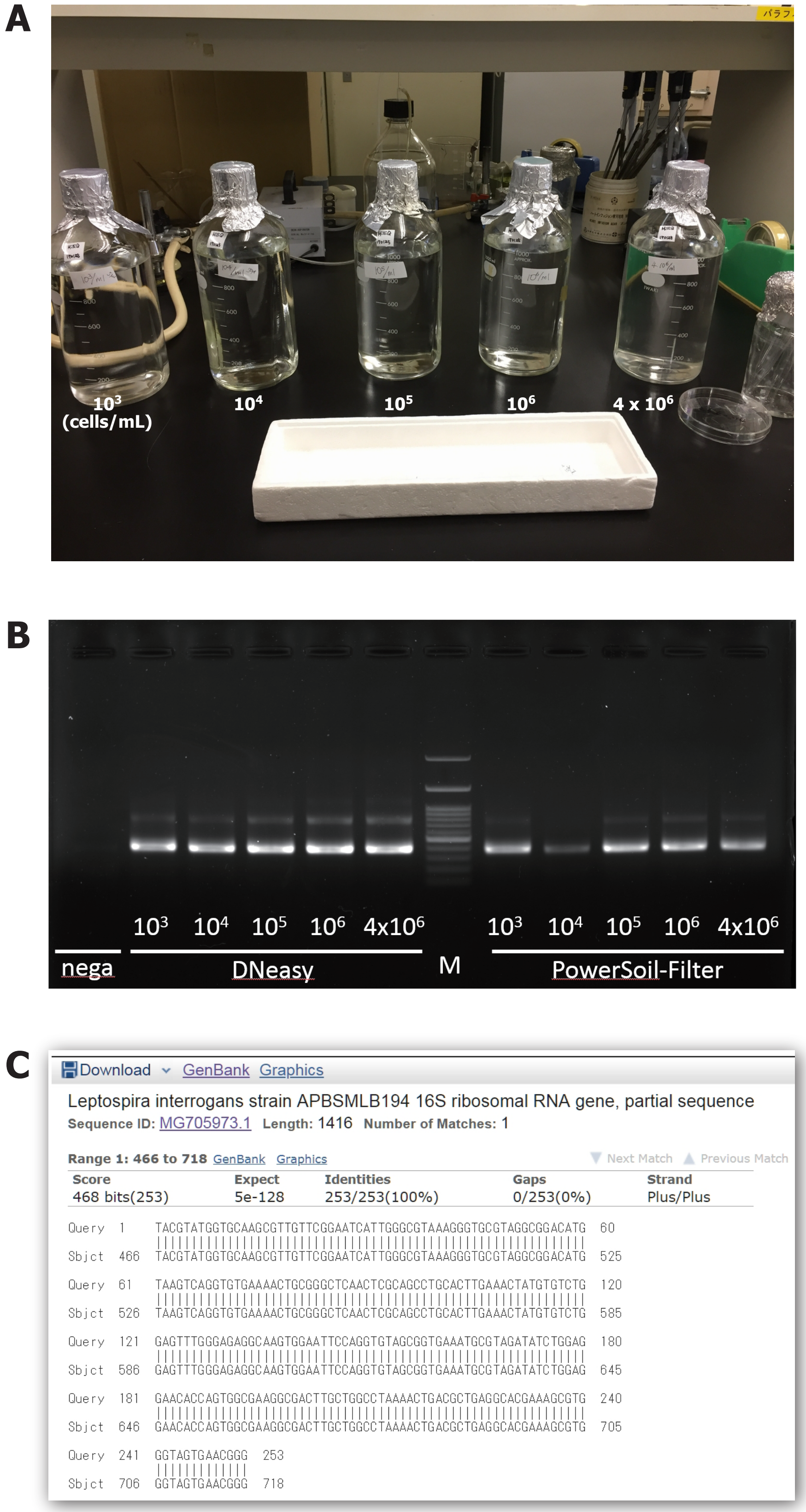

Supplementary Figure S11
